# Neighborhood Socioeconomic Status and Quality of Kidney Care: Data From Electronic Health Records

**DOI:** 10.1016/j.xkme.2021.02.008

**Published:** 2021-04-19

**Authors:** Lama Ghazi, Theresa L. Osypuk, Richard F. MacLehose, Russell V. Luepker, Paul E. Drawz

**Affiliations:** 1 Clinical and Translational Research Accelerator, Yale University, School of Medicine, New Haven, CT; 2 Division of Epidemiology and Community Health, School of Public Health, University of Minnesota, Minneapolis, MN; 3 Division of Renal Diseases and Hypertension, University of Minnesota, Minneapolis, MN

**Keywords:** Chronic kidney disease, quality of care, electronic health records, neighborhood socioeconomic status, healthcare system

## Abstract

**Rational & Objective:**

Electronic health records can be leveraged to assess quality-of-care measures in patients with chronic kidney disease (CKD). Neighborhood socioeconomic status could be a potential barrier to receiving appropriate evidence-based therapy and follow-up. We examined whether neighborhood socioeconomic status is independently associated with quality of care received by patients with CKD.

**Study Design:**

Observational study using electronic health record data.

**Setting & Participants:**

Retrospective study of patients seen at a health care system in the 7-county Minneapolis/St Paul area.

**Exposures:**

Census tract socioeconomic status measures (wealth, income, and education).

**Outcomes:**

Indicators of CKD quality of care: (1) prescription for angiotensin-converting enzyme inhibitor/angiotensin receptor blocker in patients with stage ≥ 3 CKD or stage 1 or 2 CKD with urinary albumin-creatinine ratio (UACR) > 300 mg/d, (2) UACR measurement among patients with laboratory-based CKD (estimated glomerular filtration rate < 60 mL/min/1.72 m^2^), and (3) CKD identified on the problem list or coded for at an encounter among patients with laboratory-based CKD.

**Analytic Approach:**

Multilevel Poisson regression with robust error variance with a random intercept at the census tract level.

**Results:**

Of the 16,776 patients who should be receiving an angiotensin-converting enzyme inhibitor/angiotensin receptor blocker, 65% were prescribed these medications. Among patients with laboratory-based CKD (n = 25,097), UACR was measured in 27% and CKD was identified in the electronic health record in 55%. We found no independent association between any neighborhood socioeconomic status measures and CKD quality-of-care indicators.

**Limitations:**

1 health care system and selection bias.

**Conclusions:**

We found no association of neighborhood socioeconomic status with quality of CKD care in our cohort. However, adherence to CKD guidelines is low, indicating an opportunity to improve care for all patients regardless of neighborhood socioeconomic status.

## Graphical abstract

**Figure:**
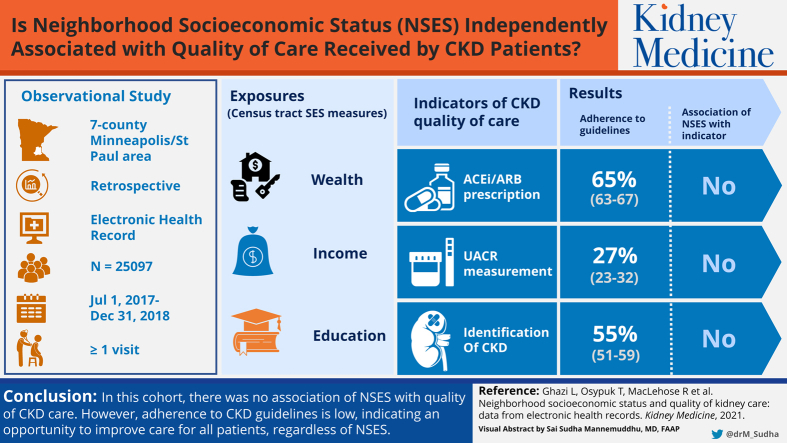

**Plain-Language Summary**

Low neighborhood socioeconomic status may be a potential barrier for patients with chronic kidney disease to receive appropriate evidence-based medical therapy and follow-up. We therefore leveraged electronic health record data from patients seen at a large health care system in Minnesota/St Paul. We found that only 35% of patients with hypertension and chronic kidney disease were not appropriately prescribed angiotensin-converting enzyme inhibitors/angiotensin receptor blockers. In patients with chronic kidney disease, urinary albumin-creatinine ratio was measured in 27% of patients and only 55% had chronic kidney disease documented in their health record. We found no difference in any quality-of-care measure by neighborhood socioeconomic status in this cohort. However, there is room to improve evidence-based quality of kidney care.

Editorial, p. 478

Chronic kidney disease (CKD) is a major public health problem in the United States; 14.8% of the US adult population has CKD. The US Surgeon General’s goal for public health for American’s citizens, Healthy People 2020, has a section focused on CKD.^1^ Goals of this policy include increasing the proportion of people with CKD who are aware that they have decreased kidney function, increasing the proportion of individuals with CKD who receive appropriate medical evaluation including evaluation for microalbuminuria, and increasing the proportion of persons with diabetes and CKD who receive recommended medical treatment with angiotensin-converting enzyme (ACE) inhibitors or angiotensin receptor blockers (ARBs).

Electronic health record (EHR) systems are increasingly adopted in the United States. Of all US hospitals, 95% currently have EHRs. Recent programs, such as the Medicare’s Physician Quality Reporting System, seek to leverage EHR systems to assess providers’ evidence-based disease management and support quality-based reimbursement strategies.^2^^,^^3^ The criteria put forth by Healthy People 2020 can be used as performance measures for CKD care in health care systems to hold providers accountable for the care they provide to patients with CKD.^4^

There is strong evidence of the association between neighborhood socioeconomic status (SES) and individuals’ health. Individuals who live in disadvantaged neighborhoods have worse self-reported health, more comorbid conditions, higher mortality rates, and even poorer dialysis outcomes than those who live in more advantaged neighborhoods.^5^, ^6^, ^7^, ^8^, ^9^ Previous studies have found that low neighborhood SES is associated with worse quality of care for cardiovascular diseases (CVDs) and diabetes.^10^, ^11^, ^12^, ^13^ However, there is a paucity of data on the role of neighborhood SES on kidney disease care, specifically early stages of CKD. The recent literature in kidney disease has started to look at the role of geographic variation and SES on pre–end-stage kidney disease (ESKD) care.^14^, ^15^, ^16^ However, there has been conflicting evidence regarding the role of neighborhood SES on pre-ESKD quality of care.^17^, ^18^, ^19^

Low neighborhood SES could be a potential barrier for patients with CKD to receive appropriate evidence-based therapy and follow-up. Our goal is to examine whether neighborhood SES is associated with quality of care among patients with CKD using EHR data from a large health care system.

## Methods

### Study Population

**Figure 1:**
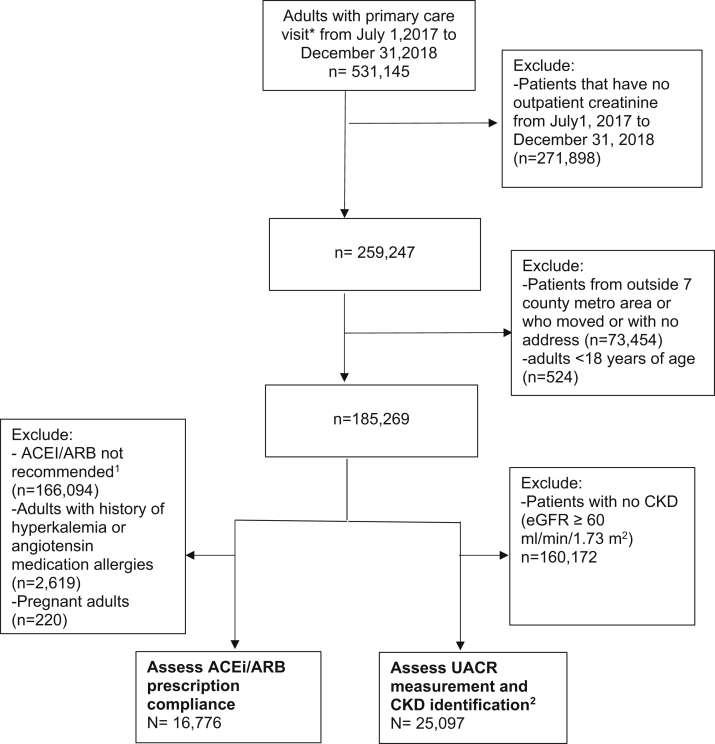
Cohort flow chart of Fairview patients from the 7-county Minneapolis/St Paul metropolitan (metro) area. ∗Primary care physician visits were defined as a completed outpatient office visits to family practice, family practice-internal medicine, internal medicine, obstetrics/gynecology, gerontology, or geriatrics clinic visit. ^1^Angiotensin-converting enzyme inhibitor (ACEI)/angiotensin receptor blocker (ARB) recommended for adults with hypertension and chronic kidney disease (CKD; stage ≥ 3 or stage 1 or 2 with urinary albumin-creatinine ratio [UACR] > 300 mg/d). ^2^CKD identification indicates CKD documented in electronic health record using *International Classification of Diseases, Ninth/Tenth Revision* codes among patients with CKD (estimated glomerular filtration rate ≤ 60 mL/min/1.73 m^2^).

Patients were identified from the EHR database of Fairview Health Services, the primary affiliate of the University of Minnesota that serves the 7-county Minneapolis/St Paul metropolitan area. The data include demographics, clinic visits, laboratory results, diagnostic codes, and geocoded addresses and census tracts of patients’ residences.^20^ The Institutional Review Board at the University of Minnesota approved this study (ID: 1502M63126). Data cannot be shared for ethical and privacy reasons. We included patients 18 years and older who had at least 1 primary care physician visit in the Fairview health system between July 1, 2017, and December 31, 2018 (Item S1), had a geocoded home address in the metropolitan area, and had at least 1 outpatient creatinine measurement during this period. We excluded patients who moved outside the metropolitan area during this period or who opted out of using their clinical data for research purposes (as shown in Fig 1).

Further exclusions were made for studying each of the 3 kidney care measures. The Kidney Care Measures section provides details on each measure. When assessing ACE-inhibitor/ARB prescription compliance by providers, we further excluded patients who had a contraindication for an ACE inhibitor/ARB: documented history of hyperkalemia, ACE-inhibitor/ARB allergies, and women who were pregnant within 1 year before or after the index creatinine level. When assessing urinary albumin-creatinine ratio (UACR) measurement and CKD documentation in the EHR, we excluded patients who had estimated glomerular filtration rates (eGFRs) ≥ 60 mL/min/1.3 m^2^ (as shown in Fig 1).

We manually abstracted clinical and laboratory data for 100 random patient charts and compared them with data pulled directly from the EHR to assess the accuracy of our EHR-based definitions*.*

### Neighborhood SES Measures

Our geographic unit of analysis was census tract, a small and relatively stable area designed to include homogeneous populations (average, 4,000 residents).^21^ We included 3 measures to operationalize tract-level SES: median value of owner-occupied housing units, percentage of residents older than 25 years with a bachelor’s degree or higher, and median household income as identified from the American Community Survey 5-year data (2008-2012). These measures reflect wealth, education, and income of the tract.^22^, ^23^, ^24^ Low- and high-SES census tracts were defined as belonging to the first and fourth quartiles of the distribution of each of their respective measures using census data of the 7-county metropolitan area. A patient was considered to be living in a low-SES census tract (quartile 1 of the distribution of each SES) if the tract median value of owner-occupied housing units was <$165,200, tract percent of residents older than 25 years with a bachelor’s degree or higher was <20.4%, or tract median household income was <$35,935. We linked each patient’s residence to their census tract and tract characteristics.

### Kidney Care Measures

All creatinine measures were traceable to an isotope-dilution mass spectrometry reference measurement. eGFR was calculated using the CKD Epidemiology Collaboration (CKD-EPI) creatinine equation.^25^ The last outpatient creatinine level measured between July 1, 2017, and December 31, 2018 (index creatinine/eGFR), was used for all analyses. We assessed 3 measures of kidney care (as shown in Fig 1): prescription of ACE inhibitor/ARB by providers, UACR measurement, and identification of CKD in the EHR.

#### ACE-Inhibitor/ARB Prescription by Providers

ACE-inhibitor/ARB prescription by providers was assessed by querying the medication list (includes active orders and documented home or self-reported medications) between July 1, 2017, and December 31, 2018, and up to 90 days after the index creatinine level. At each clinic visit, providers are encouraged to reconcile medications and document any changes in the EHR. We identified 16,776 patients who should be receiving an ACE inhibitor/ARB according to the 2017 American College of Cardiology/American Heart Association (ACC/AHA) *Guidelines for the Prevention, Detection, Evaluation and Management of High Blood Pressure in Adults*; the presence of hypertension and CKD (stage ≥ 3 or stage 1 or 2 with UACR > 300 mg/d) with no documentation of a contraindication for an ACE inhibitor/ARB.^26^

#### UACR Measurement and CKD Documentation in the EHR

UACR measurement was assessed by querying outpatient laboratories between July 1, 2017, and December 31, 2018, and up to 90 days after the index creatinine level. We identified 25,097 patients with CKD, defined as having an eGFR < 60 mL/min/1.3 m^2^, for whom it is recommended to test for albuminuria using UACR.^27^^,^^28^ UACR measurement adherence was defined as ever having had a UACR measured during the period.

We also assessed whether CKD was documented in the patients’ EHR (problem list, medical history, diagnosis, or procedure code), which was defined as having at least 2 *International Classification of Diseases, Ninth/Tenth Revision* (*ICD-9/10*) codes for CKD, ESKD, dialysis, or kidney transplantation documented before and up until 90 days after the date of the index creatinine level among patients with laboratory-based CKD. This was assessed among the 25,097 patients with CKD. A diagnosis of CKD could reflect both physicians’ awareness of CKD and in turn could be a proxy of patients’ awareness of having impaired kidney function. For all models, kidney care measures were each considered separately as dichotomous outcomes.

### Variables

All data were obtained from the EHR, including sex, race, and insurance (Item S1). Age, smoking status, and body mass index were defined using the last value before or at the time of the index creatinine level. Comorbid conditions (CVD, stroke, cancer, hyperlipidemia, diabetes, and hypertension) were considered present if at least 2 *ICD-9/10* codes for that condition were present at or before the date of the index creatinine level.^29^

### Statistical Analysis

We evaluated the potential sparsity of data by using contingency tables to assess the number of participants for each kidney care measure by tract SES.^30^^,^^31^ Descriptive statistics were used to assess the characteristics of patients who are eligible to be receiving ACE inhibitors/ARBs, those with and without UACR measurement, and those with and without identified CKD. Additionally, we looked at the percentage of patients who received appropriate kidney care by quartile of neighborhood SES and additionally stratified by race. Pearson correlation coefficient was used to assess the association among the census tract SES variables.

We used regression models with a random intercept for census tract level to estimate the tract-level SES association (low SES = quartile 1 vs high SES = quartile 4, for each of the 3 neighborhood SES measures) with kidney care measures. Because all kidney care measures were common outcomes, we estimated the prevalence ratio (PR) and 95% CI using Poisson regression with robust error variance.^32^, ^33^, ^34^ To estimate the association of neighborhood SES (low SES = quartile 1 vs high SES = quartile 4) with ACE-inhibitor/ARB prescription compliance by providers, we fit these models: model 1, crude; model 2, model 1 plus age, sex, race, obesity, smoking, and insurance status; and model 3, model 2 plus history of CVD, stroke, cancer, hyperlipidemia, and diabetes. We assessed whether there was effect modification on the multiplicative scale by race and sex. To estimate the association of neighborhood SES (low SES = quartile 1 vs high SES = quartile 4) with UACR measurement and CKD identification in the EHR, we fit similar models to those described and added index eGFR to model 3. We assessed whether there was effect modification on the multiplicative scale by sex, race, hypertension, and diabetes history for these 2 outcomes.

Because we included only patients who had their kidney function measured and met our inclusion criteria, results are potentially subject to selection bias (selection into the study may differ based on quality indicators and neighborhood SES). We quantitatively assessed the impact of this selection bias by estimating a selection bias–adjusted PR.^35^ We multiplied each cell in the 2×2 table (exposure: high vs low neighborhood SES; outcome: ACE-inhibitor/ARB prescription compliance by providers vs not/UACR measured vs not/CKD identified in EHR vs not) with a selection factor (probability of a patient being included in the cohort given kidney care measured received and tract characteristics) to estimate the adjusted PR. A range of selection factors were subjectively used.^35^ For further details see Tables S1 and S2.

Sensitivity analysis included the following: using a restrictive definition of CKD (eGFR < 45 mL/min/1.3 m^2^ per year, as opposed to the cut point of eGFR < 60 mL/min/1.3 m^2^) to assess UACR measurement performance and CKD identified in EHR, changing the 18-month study period (July 1, 2017, to December 31, 2018) to a 36-month study period (December 31, 2015, to December 31, 2018), and assessing broader eligibility criteria to be prescribed ACE inhibitors/ARBs; that is, eligibility will be based on the ACC/AHA guidelines and/or the American Diabetes Association guidelines (recommended for patients with diabetes, hypertension, and albuminuria [UACR > 30 mg/d]). Statistical analyses were conducted using R (R Foundation for Statistical Computing) and Stata (StataCorp).^36^^,^^37^

## Results

### Cohort Characteristics

We identified 531,145 patients with a primary care physician visit in our query; 185,269 patients had creatinine measured and resided in the 7-county metropolitan area. Of those, 16,776 were eligible to be receiving an ACE inhibitor/ARB and 25,097 had CKD (as shown in Fig 1). Baseline characteristics of these patient populations are shown in Tables 1, 2, and S3 to S5. Tract income was moderately correlated with tract wealth and education (*r*=0.30 and *r*=0.33, respectively). However, tract wealth was more strongly correlated with tract education (*r*=0.68).

**Table 1:** Characteristics of Patients by Neighborhood SES in Cohort Used to Determine Angiotensin Prescription Compliance

	Low SES (quartile 4 of median value of owner-occupied housing units [≥$231,300]) (n = 1,607)	High SES (quartile 1 of median value of owner-occupied housing units [<$165,200]) (n = 6,825)
eGFR, mL/min/1.3 m^2^	46.3 ± 18.2	48.1 ± 14.5
UACR, mg/d^a^	60.7 [15.6-480.0]	34.7 [11.1-289.9]
Albuminuria (>300 mg/d)^a^	193 (33%)	538 (25%)
Individual-level demographic characteristics		
Age, y	70.5 ± 15.2	73.3 ± 12.9
Male sex	617 (38%)	3,126 (46%)
Black race	229 (14%)	187 (3%)
Individual social characteristics		
Smoker	809 (50%)	3,327 (49%)
Medicaid or Medicare	332 (21%)	1,101 (16%)
Vital signs		
Systolic BP, mm Hg	133.4 ± 20.2	131.4 ± 19.7
Diastolic BP, mm Hg	75.7 ± 12.5	74.3 ± 11.8
Medical history		
Diabetes	706 (44%)	2,217 (33%)
Obese (BMI ≥ 30 kg/m^2^)	706 (46%)	2,667 (42%)
Cardiovascular disease	625 (39%)	2,996 (44%)
Stroke	169 (11%)	788 (12%)
Hyperlipidemia	1,199 (75%)	5,367 (79%)
Cancer	198 (12%)	1,121 (16%)

	Low SES (quartile 4 of % aged > 25 y with ≥bachelor’s degree [≥$48.1%]) (n = 1,945)	High SES (quartile 1 of % aged > 25 y with ≥bachelor’s degree [<20.4%]) (n = 4,600)
eGFR, mL/min/1.3 m^2^	47.2 ± 18.4	48.2 ± 14.4
UACR, mg/d	53.3 [13.7-424.7]	34.9 [11.6-305.8]
Albuminuria (>300 mg/d)^b^	236 (31%)	360 (25%)
Individual-level demographic characteristics		
Age, y	70.0 ± 14.4	73.5 ± 12.9
Male sex	780 (40%)	2,105 (46%)
Black race	209 (11%)	153 (3%)
Individual social characteristics		
Smoker	1,004 (52%)	2,250 (49%)
Medicaid or Medicare	418 (22%)	728 (16%)
Vital signs		
Systolic BP, mm Hg	133.4 ± 20.2	131.4 ± 19.7
Diastolic BP, mm Hg	75.7 ± 12.5	74.3 ± 11.8
Medical history		
Diabetes	877 (45%)	1,447 (32%)
Obese (BMI ≥ 30 kg/m^2^)	942 (51%)	1,663 (39%)
Cardiovascular disease	759 (39%)	2,059 (45%)
Stroke	206 (11%)	503 (11%)
Hyperlipidemia	1,480 (76%)	3,571 (78%)
Cancer	231 (12%)	766 (17%)

	**Low SES (quartile 4 of median household income [≥$62,343])** **(n = 1,807)**	**High SES (quartile 1 of median household income [<$35,935])** **(n = 8,664)**

eGFR, mL/min/1.3 m^2^	47.4 ± 15,8	48.2 ± 14.7
UACR, mg/d^c^	43.4 [12.3-353.8]	34.0 [11.4-288.7]
Albuminuria (>300 mg/d)^c^	178 (29%)	756 (25%)
Individual-level demographic characteristics		
Age, y	71.9 ± 13.4	72.7 ± 13.1
Male sex	779 (43%)	3,758 (43%)
Black race	205 (11%)	303 (4%)
Individual social characteristics		
Smoker	921 (51%)	4,323 (50%)
Medicaid or Medicare	300 (16%)	1,502 (18%)
Vital signs		
Systolic BP, mm Hg	132.5 ± 20.2	131.8 ± 19.6
Diastolic BP, mm Hg	74.9 ± 11.5	74.4 ± 11.8
Medical history		
Diabetes	677 (38%)	3,074 (36%)
Obese (BMI ≥ 30 kg/m^2^)	736 (44%)	3,672 (45%)
Cardiovascular disease	768 (43%)	3,725 (43%)
Stroke	198 (11%)	956 (11%)
Hyperlipidemia	1,347 (75%)	6,828 (79%)
Cancer	279 (15%)	1,394 (16%)

*Note:* Nonpregnant adults with hypertension and chronic kidney disease (stage ≥ 3 or stage 1 or 2 with UACR > 300 mg/d). Values expressed as mean ± standard deviation, median [interquartile range], or number (percent). Abbreviations and Definitions: BMI, body mass index; BP, blood pressure; cardiovascular disease, includes congestive heart failure, acute myocardial infarction, ischemic heart disease, and peripheral vascular disease; eGFR, estimated glomerular filtration rate; SES, socioeconomic status; UACR, urinary albumin-creatinine ratio.

a Number (percent) with albuminuria is among those who had available UACR measurements. Among those with low SES (median value of owner-occupied housing units), UACR was measured in 594/1,607 patients and 193 of those had albuminuria [UACR > 300 mg/d]. Among those with high SES, UACR was measured in 2,162/6,825 patients and 538 of those had albuminuria.

b Number (percent) with albuminuria is among those who had available UACR measurements. Among those with low SES (percent aged > 25 years with bachelor’s degree or higher), UACR was measured in 751/1,945 patients and 236 of those had albuminuria [UACR > 300 mg/d]. Among those with high SES, UACR was measured in 1,413/4,600 patients and 360 of those had albuminuria.

c Number (percent) with albuminuria is among those who had available UACR measurements. Among those with low SES (household income), UACR was measured in 620/1,807 patients and 178 of those had albuminuria (UACR > 300 mg/d). Among those with high SES, UACR was measured in 3,052/8,664 patients and 756 of those had albuminuria.

**Table 2:** Characteristics of Patients by Neighborhood SES in Cohort Used to Determine UACR Measurement and Chronic Kidney Disease Identification in the Electronic Health Record

	Low SES (quartile 4 of median value of owner-occupied housing units [≥$231,300]) (n = 2,375)	High SES (quartile 1 of median value of owner-occupied housing units [<$165,200]) (n = 10,611)
eGFR, mL/min/1.73 m^2^	44.6 ± 14.1	47.9 ± 11.6
UACR, mg/d^a^	37.5 [12.5-190.4]	25.9 [9.8-293.8]
Albuminuria (>300 mg/d)^a^	143 (21%)	407 (16%)
Individual-level demographic characteristics		
Age, y	69.2 ± 15.4	70.9 ± 13.7
Male sex	892 (38%)	4,659 (44%)
Black race	292 (12%)	245 (2%)
Individual social characteristics		
Smoker	1,218 (51%)	4,886 (46%)
Medicaid or Medicare	474 (20%)	1,548 (15%)
Vital signs		
Systolic BP, mm Hg	131.6 ± 20.6	129.6 ± 19.7
Diastolic BP, mm Hg	75.2 ± 15.6	74.3 ± 11.7
Medical history		
Diabetes	1,925 (81%)	7,938 (75%)
Obese (BMI ≥ 30 kg/m^2^)	902 (38%)	2,842 (27%)
Cardiovascular disease	972 (44%)	3,722 (38%)
Stroke	868 (37%)	3,888 (37%)
Hyperlipidemia	237 (10%)	1,030 (10%)
Cancer	1,564 (66%)	7.217 (68%)

	**Low SES (quartile 4 of % aged > 25 y with ≥bachelor’s degree [≥$48.1%])** **(n = 2,795)**	**High SES (quartile 1 of % aged > 25 y with ≥bachelor’s degree [<20.4%])** **(n = 7,173)**

eGFR, mL/min/1.73 m^2^	45.2 ± 13.5	48.0 ± 11.4
UACR, mg/d^b^	35.5 [11.8-202.7]	25.5 [10.4-106.6]
Albuminuria (>300 mg/d)^b^	186 (22%)	262 (16%)
Individual-level demographic characteristics		
Age, y	68.7 ± 14.7	71.2 ± 13.8
Male sex	1,086 (39%)	3,148 (44%)
Black race	254 (9%)	189 (3%)
Individual social characteristics		
Smoker	1,480 (53%)	3,265 (46%)
Medicaid or Medicare	558 (20%)	1,036 (15%)
Vital signs		
Systolic BP, mm Hg	131.7 ± 20.7	129.6 ± 19.9
Diastolic BP, mm Hg	74.9 ± 12.6	74.3 ± 11.7
Medical history		
Diabetes	2,260 (81%)	5,335 (74%)
Obese (BMI ≥ 30 kg/m^2^)	1,080 (39%)	1,844 (26%)
Cardiovascular disease	1,245 (48%)	2,290 (35%)
Stroke	1,002 (36%)	2,666 (37%)
Hyperlipidemia	273 (10%)	661 (9%)
Cancer	1,891 (68%)	4,817 (67%)

	**Low SES (quartile 4 of median household income [≥$62,343])** **(n = 2,723)**	**High SES (quartile 1 of median household income [<$35,935])** **(n = 13,104)**

eGFR, mL/min/1.73 m^2^	46.2 ± 13.0	47.6 ± 11.8
UACR, mg/d^c^	31.8 [10.9-153.2]	26.3 [10.3-120.1]
Albuminuria (>300 mg/d)^c^	138 (19%)	564 (16%)
Individual-level demographic characteristics		
Age, y	70.0 ± 14.1	70.7 ± 13.9
Male sex	1,154 (42%)	5,453 (42%)
Black race	260 (10%)	374 (3%)
Individual social characteristics		
Smoker	1,333 (49%)	6,234 (48%)
Medicaid or Medicare	413 (15%)	2,106 (16%)
Vital signs		
Systolic BP, mm Hg	130.9 ± 20.2	130.2 ± 19.7
Diastolic BP, mm Hg	74.7 ± 11.8	74.4 ± 11.9
Medical history		
Diabetes	2,132 (78%)	10,073 (77%)
Obese (BMI ≥ 30 kg/m^2^)	866 (32%)	3,859 (29%)
Cardiovascular disease	996 (40%)	4,988 (41%)
Stroke	1,024 (38%)	4,823 (37%)
Hyperlipidemia	262 (10%)	1,257 (10%)
Cancer	1,798 (66%)	9,041 (69%)

*Note:* Have CKD indicates eGFR < 60 mL/min/1.73 m^2^. Values expressed as mean ± standard deviation, median [interquartile range], or number (percent).

Abbreviations and Definitions: BMI, body mass index; BP, blood pressure; cardiovascular disease, includes congestive heart failure, acute myocardial infarction, ischemic heart disease, and peripheral vascular disease; CKD, chronic kidney disease; eGFR, estimated glomerular filtration rate; SES, socioeconomic status; UACR, urinary albumin-creatinine ratio.^a^. ^b^. ^c^

### Kidney Care Measures and Neighborhood SES

We had sufficient numbers of patients in each SES strata for each kidney care measure. Our analysis found that quality of kidney care was moderate to low depending on which of the measures was examined. ACE-inhibitor/ARB prescription adherence was 65% in the overall cohort. UACR measurement performance and CKD identification in the EHR in patients with CKD were 27% and 55%, respectively. The distribution of all kidney care measures was similar across quartiles of all neighborhood SES measures and by race (Table S6). Characteristics of patients by kidney care measures are shown in Tables S3 to S5. Patients who were prescribed ACE inhibitors/ARBs were more likely to have comorbid conditions than those who were not. Those who had UACR measured versus not were older, more likely to be Black, had more comorbid conditions, and a higher percentage lived in lower neighborhood SES. As for patients who had CKD identified in the EHR versus not, they were older, more likely to be male and Black and have more comorbid conditions, and a higher percentage lived in lower neighborhood SES.

Living in a low neighborhood SES (quartile 1) compared with high neighborhood SES (quartile 4; wealth, education, and income) was not associated with ACE-inhibitor/ARB prescription compliance by providers, UACR measurement, or CKD identification in the EHR after adjusting for demographics and clinical characteristics (Table 3, Table 4, Table 5, model 3). However, belonging to the second quartile of wealth was associated with 1.13 times the prevalence (95% CI, 1.04-1.22] of UACR measurement relative to belonging to high neighborhood SES wealth. Similarly, belonging to quartile 2 or 3 of tract SES, education was associated with 1.10 times the prevalence of UACR measurement than living in high SES tracts (1.10; 95% CI, 1.00-1.17) for quartile 2and 1.10 (95% CI, 1.02-1.19] for quartile 3.

**Table 3:** Multilevel Regression Model for the Association of Tract-Level SES With Angiotensin Medication Prescription Compliance by Providers

	Model 1 PR (95% CI)	Model 2 PR (95% CI)	Model 3 PR (95% CI)
Median value of owner-occupied housing units is the measure of SES			
High SES tract, Q4 (n = 6,825)	1.00	1.00	1.00
Q3 (n = 6,144)	0.99 (0.96-1.02)	0.99 (0.96-1.02)	0.99 (0.96-1.01)
Q2 (n = 2,196)	1.04 (1.00-1.08)	1.03 (0.99-1.07)	1.01 (0.98-1.05)
Low SES tract, Q1 (n = 1,607)	0.98 (0.92-1.05)	0.97 (0.91-1.04)	0.96 (0.91-1.03)
Percent of residents aged > 25 y with ≥bachelor’s degree			
High SES tract, Q4 (n = 4,600)	1.00	1.00	1.00
Q3 (n = 5,668)	1.00 (0.97-1.04)	1.02 (0.98-1.07)	0.99 (0.96-1.03)
Q2 (n = 4,560)	1.03 (0.99-1.07)	1.03 (0.99-1.06)	1.01 (0.98-1.04)
Low SES tract, Q1 (n = 1,945)	1.04 (1.00-1.09)	1.02 (0.98-1.07)	1.01 (0.97-1.05)
Median household income			
High SES tract, Q4 (n = 8,664)	1.00	1.00	1.00
Q3 (n = 3,956)	0.97 (0.94-1.01)	0.98 (0.95-1.02)	0.98 (0.95-1.01)
Q2 (n = 2,334)	0.99 (0.95-1.03)	0.99 (0.95-1.03)	0.99 (0.95-1.03)
Low SES tract, Q1 (n = 1,807)	0.96 (0.92-1.00)	0.96 (0.93-1.00)	0.97 (0.94-1.02)

*Note:* Adults (nonpregnant patients) with hypertension and chronic kidney disease (stage ≥ 3 or stage 1 or 2 with UACR > 300 mg/d) should be taking ACEis and ARBs. ACEi/ARB prescription compliance by providers: yes if recommended ACEi/ARB prescribed. Median value of owner-occupied housing units: high SES (Q4), ≥$231,300; Q3, $188,100 to $231,300; Q2, $165,200 to $188,100; low SES (Q1), <$165,200; percent older than 25 years with a Bachelor’s degree or higher, high SES (Q4), ≥48.1%; Q3, 34.1% to 48.1%; Q2, 20.4% to 34.1%; low SES (Q1), <20.4%; median household income: high SES (Q4), ≥$62,343; Q3, $47,379 to $62,343; Q2, $35,935 to $47,379; low SES (Q1), <$35,935. Model 1, crude; model 2, age, sex, race, obesity, smoking, and insurance status; model 3, model 2 plus history of cardiovascular disease, stroke, cancer, hyperlipidemia, and diabetes. *P* value for interaction of wealth with race (*P* = 0.69) and with sex (*P* = 0.01); of education with race (*P* = 0.04) and with sex (*P* = 0.11); and of income with race (*P* = 0.47) and with sex (*P* = 0.62).

Abbreviations: ACEi, angiotensin-converting enzyme inhibitor; ARB, angiotensin receptor blocker; PR, prevalence ratio; Q, quartile; SES, socioeconomic status; UACR, urinary albumin-creatinine ratio.

**Table 4:** Multilevel Regression Model for the Association of Tract Level SES With Urinary Albumin-Creatinine Ratio Measurement

	Model 1 PR (95% CI)	Model 2 PR (95% CI)	Model 3 PR (95% CI)
Median value of owner-occupied housing units is the measure of SES			
High SES tract, Q4 (n = 10,611)	1.00	1.00	1.00
Q3 (n = 8,940)	1.19 (1.11-1.28)	1.15 (1.07-1.23)	1.07 (0.99-1.13)
Q2 (n = 3,164)	1.35 (1.23-1.48)	1.27 (1.16-1.39)	1.13 (1.04-1.22)
Low SES tract, Q1 (n = 2,375)	1.22 (1.09-1.36)	1.16 (1.04-1.30)	1.01 (0.91-1.12)
Percent of residents aged >25 y with ≥bachelor’s degree			
High SES tract, Q4 (n = 7,173)	1.00	1.00	1.00
Q3 (n = 8,450)	1.21 (1.11-1.31)	1.15 (1.06-1.25)	1.10 (1.00-1.17)
Q2 (n = 6,673)	1.30 (1.20-1.42)	1.23 (1.14-1.34)	1.10 (1.02-1.19)
Low SES tract, Q1 (n = 2,795)	1.34 (1.22-1.48)	1.25 (1.14-1.38)	1.07 (0.98-1.17)
Median household income			
High SES tract, Q4 (n = 13,104)	1.00	1.00	1.00
Q3 (n = 5,857)	1.02 (0.95-1.08)	0.99 (0.92-1.07)	0.98 (0.92-1.05)
Q2 (n = 3,391)	0.98 (0.88-1.09)	0.95 (0.86-1.05)	0.92 (0.84-1.00)
Low SES tract, Q1 (n = 2,723)	0.97 (0.87-1.08)	0.96 (0.86-1.07)	0.96 (0.87-1.06)

*Note:* UACR measured in patients with chronic kidney disease (eGFR < 60 mL/min/1.73 m^2^). Median value of owner-occupied housing units: high SES (Q4), ≥$231,300; Q3, $188,100 to $231,300; Q2, $165,200 to $188,100; low SES (Q1), <$165,200; percent older than 25 years with bachelor’s degree or higher: high SES (Q4), ≥48.1%; Q3, 34.1% to 48.1%; Q2, 20.4% to 34.1%; low SES (Q1), <20.4%; Median household income: high SES (Q4), ≥$62,343; Q3, $47,379 to $62,343; Q2, $35,935 to $47,379; low SES (Q1), <$35,935. Model 1, crude; model 2, age, sex, race, obesity, smoking, and insurance status; model 3, model 2 plus history of cardiovascular disease, stroke, cancer, hyperlipidemia, diabetes, hypertension, and index eGFR. *P* value for interaction of wealth with race (*P* = 0.12), with sex (*P* = 0.11), hypertension (*P =* 0.05), and diabetes (*P* = 0.21); of education with race (*P* = 0.52), with sex (*P* = 0.33), hypertension (*P* = 0.54), and diabetes (*P* = 0.17); and of income with race (*P* = 0.21), with sex (*P* = 0.46), hypertension (*P* = 0.23), and diabetes (*P* = 0.44).

Abbreviations: eGFR, estimated glomerular filtration rate; PR, prevalence ratio; Q, quartile; SES, socioeconomic status, UACR, .urinary albumin-creatinine ratio

**Table 5:** Multilevel Regression Model for the Association of Tract-Level SES With CKD Identification

	Model 1 PR (95% CI)	Model 2 PR (95% CI)	Model 3 PR (95% CI)
Median value of owner-occupied housing units is the measure of SES			
High SES tract, Q4 (n = 10,611)	1.00	1.00	1.00
Q3 (n = 8,940)	1.11 (1.07-1.14)	1.09 (1.06-1.12)	1.03 (1.00-1.06)
Q2 (n = 3,164)	1.14 (1.09-1.21)	1.11 (1.06-1.17)	1.03 (0.99-1.08)
Low SES tract, Q1 (n = 2,375)	1.15 (1.11-1.20)	1.11 (1.07-1.16)	1.02 (0.98-1.06)
Percent aged > 25 y with ≥bachelor’s degree			
High SES tract, Q4 (n = 7,173)	1.00	1.00	1.00
Q3 (n = 8,450)	1.08 (1.04-1.12)	1.06 (1.03-1.10)	1.02 (0.99-1.05)
Q2 (n = 6,673)	1.12 (1.08-1.16)	1.09 (1.06-1.14)	1.02 (0.98-1.05)
Low SES tract, Q1 (n = 2,795)	1.16 (1.10-1.22)	1.13 (1.08-1.19)	1.03 (0.98-1.07)
Median household income			
High SES tract, Q4 (n = 13,104)	1.00	1.00	1.00
Q3 (n = 5,857)	1.07 (1.03-1.13)	1.04 (1.00-1.07)	1.01 (0.97-1.04)
Q2 (n = 3,391)	1.09 (1.06-1.14)	1.07 (1.03-1.10)	1.02 (0.98-1.05)
Low SES tract, Q1 (n = 2,723)	1.07 (1.01-1.10)	1.03 (0.98-1.08)	1.01 (0.97-1.06)

*Note:* CKD identification indicates CKD documented in electronic health record using *International Classification of Diseases, Ninth/Tenth Revision* codes among patients with CKD (eGFR ≤ 60 mL/min/1.73 m^2^). Median value of owner-occupied housing units: high SES (Q4), ≥$231,300; Q3, $188,100 to $231,300; Q2, $165,200 to $188,100; low SES (Q1), <$165,200; percent older than 25 years with bachelor’s degree or higher: high SES (Q4), ≥48.1%; Q3, 34.1% to 48.1%; Q2, 20.4% to 34.1%; low SES (Q1), <20.4%; median household income: high SES (Q4), ≥$62,343; Q3, $47,379 to $62,343; Q2, $35,935 to $47,379; low SES (Q1), <$35,935. Model 1, crude; model 2, age, sex, race, obesity, smoking, and insurance status; model 3: model 2 plus history of cardiovascular disease, stroke, cancer, hyperlipidemia, diabetes, hypertension, and index eGFR. *P* value for interaction of wealth with race (*P* = 0.02), with sex (*P* = 0.08), hypertension (*P* = 0.001), and diabetes (*P* = 0.04); of education with race (*P* = 0.24), with sex (*P* = 0.14), hypertension (*P* = 0.03), and diabetes (*P* = 0.22); and of income with race (*P* = 0.16), with sex (*P* = 0.35), hypertension (*P* = 0.36), and diabetes (*P* = 0.03).

Abbreviations: CKD, chronic kidney disease; eGFR, estimated glomerular filtration rate; PR, prevalence ratio of chronic kidney disease for individual in lowsocioeconomic status tract vs high socioeconomic status tract; Q, quartile; SES, socioeconomic status.

### Bias and Sensitivity Analysis

In our bias analyses, over a range of selection probabilities, we found that the selection bias–adjusted PR for the association of tract SES (low SES = quartile 1 vs high SES = quartile 4) and kidney care measures compared with the observed PR varied depending on selection probabilities (Tables S1 and S2). For example, the PR for the crude association of neighborhood SES wealth (low SES = quartile 1 vs high SES = quartile 4) with ACE-inhibitor/ARB prescription compliance by providers was 0.98; after adjusting for selection bias, PRs range between 0.69 and 1.11.

Our results were consistent across all sensitivity analyses, including a more restrictive definition of CKD, a cohort covering a 36-month period, and using the ACC/AHA and American Diabetes Association guidelines to determine ACE-inhibitor/ARB eligibility (Table S7-S9). Because sex and race were shown to be effect modifiers of the association of neighborhood SES with angiotensin medication prescription compliance, we studied the association in males, females, Blacks, and non-Blacks separately. Results are shown in Tables S10 and S11. We looked at the association of neighborhood SES with CKD being identified in the EHR by identified effect modifiers (hypertension, diabetes, and race) and results are shown in Table S12**.** Of note, compared with patients included in our cohort, those excluded were healthier and younger but had similar characteristics to the population in the Twin Cities metropolitan area (Tables S13 and S14). Data adjudicated from chart manual review for *ICD-9/10* codes and medications were consistent with clinic notes.

## Discussion

We found that there is room to improve the evidence-based quality of CKD care. Among eligible patients with hypertension and CKD, 35% were not prescribed ACE inhibitors/ARBs by providers. In patients with CKD, UACR was not measured in 73% of patients and 45% of patients did not have CKD documented in the EHR. Neighborhood SES (low SES = quartile 1 vs high SES = quartile 4) was not associated with kidney care measures after adjusting for demographics and clinical characteristics. The lack of association in this population may be attributed to uncontrolled confounders, selection bias, and that certain variables (eg, diabetes and hypertension) may mediate these associations.

There has been conflicting evidence of the association of neighborhood SES and county SES and pre-ESKD care. Some studies reported lower quality of pre-ESKD nephrology care in urban counties, counties with low educational attainment (less than high school), and in zip codes with low levels of median household income.^17^^,^^18^ However, Plantinga et al^19^ did not find an association between census tract poverty level and pre-ESKD care. Our study is the first to assess quality of care by neighborhood SES in all patients with CKD using Healthy People 2020 relevant metrics, and we found no independent association of neighborhood SES (low SES = quartile 1 vs high SES = quartile 4) with kidney care measures.

Paradoxically, we found that patients belonging to quartiles 2 and 3 of neighborhood SES for education and wealth had a greater prevalence of UACR measurement compared with the quartile 4 (high SES tract). This was not observed when comparing the first (low SES) versus fourth quartile (high SES) of tract SES for wealth or education. The reason behind this observation is unclear and could simply be random error. We also observed that patients with no history of hypertension and White patients living in low-wealth SES tracts (quartile 1) were more likely to have CKD identified in the EHR relative to those living in high SES tracts (quartile 4). This same association was not observed in patients with hypertension or in Black patients (Tables S10-S12). We can only speculate that these paradoxical observations might be attributable to a combination of uncontrolled confounders and selection bias. Stronger study designs, such as recruiting a population-based sample of patients and assessing their kidney function along with collecting individual-level SES, are needed to further explain these associations.

Our results should be interpreted with caution because they are only applicable to patients who had their creatinine level measured and received primary care from within the health system. Patients excluded from our cohort were healthier and younger than those included in the analysis (Table S13). However, the distribution of patients by tract SES was similar. To account for this selection, we estimated a range of selection bias–adjusted PRs. The selection bias–adjusted PRs of the associations of kidney care received with neighborhood SES in the Fairview population reflect a wide range of estimates compared with the crude PR. This again indicates the need to recruit a population-based sample to further understand the role of neighborhood SES on kidney care.

Previous studies have looked at overall quality of care provided to patients with CKD. A study of 11,774 patients with CKD (eGFR, 15-60 mL/min/1.3 m^2^) with an averageage of 73 years from a multispecialty primary care group practice in Massachusetts found low rates of adherence to many CKD practice guidelines, including annual urinary protein quantification (30%), receipt of appropriate ACE-inhibitor/ARB medications (75%), and documentation of CKD on their problem list (24%).^38^ An analysis of Southern California Kaiser Permanente reported that over a 12-month period, only 79% of patients with CKD had it coded in their EHR. They reported that in patients with prevalent CKD, 79% had proteinuria assessed and 84% had ACE inhibitors/ARBs on their medication list.^39^ Additionally, in a large managed care cohort of 10,000 patients with CKD, physician documentation of CKD with *ICD* codes was only 14%.^40^ The US Renal Data System reports that only 26% of patients with CKD received medical evaluation with serum creatinine level, lipid levels, and microalbuminuria in 2007, and only 67% of individuals with diabetes and CKD received ACE inhibitors/ARBs.^1^ Trends observed in these studies are consistent with our findings, especially with very low rates of UACR measurement. Because CKD identification in the EHR in our study was only 55%, the higher rate of ACE-inhibitor/ARB prescription compliance by providers was likely due in part to management of CVD or diabetes in these patients.

Our study has several limitations. First, we studied a single health care system in Minnesota, which may not be representative of Minnesota or other states. However, we have shown that patients included in this analysis are similar to the population in the Twin Cities metropolitan area (Table S14).

Second, there are limitations to the current EHR data quality, including lack of individual-level SES measures and coarse race measures. Improving data capture and improving the quality of demographic and individual socioeconomic data collected in EHRs will help overcome these limitations.

Third, misclassification is a concern because we used *ICD-9/10* codes to determine comorbid conditions and medication lists (including active orders and documented home/self-reported) to determine medications only from EHR queries and not clinic notes. These may differentially affect patients of certain SES and/or neighborhood SES. To minimize misclassification bias by *ICD-9/10* codes, we used at least 2 *ICD-9/10* codes to confirm diagnoses. We also adjudicated 100 patient charts and found that *ICD-9/10* code diagnoses and medications were consistent with clinic notes.

Fourth, we did not evaluate patient adherence to ACE-inhibitor/ARB therapy or assess their awareness of having CKD. Medication adherence is particularly important in the context of chronic diseases, especially because 50% of patients do not take their medications as prescribed.^41^

Fifth, we used only a 1-time eGFR measurement to determine CKD prevalence. However, our findings derived from sensitivity analyses were consistent when using a more restrictive definition of CKD and models assessing UACR measurement and CKD identification in the EHR were adjusted for eGFR.

Sixth, we did not look at other aspects of CKD care such as treatments to reduce patients’ risk for CVD and measures to address anemia, metabolic acidosis, bone and mineral disorders, and early referral to nephrologists.^42^ This is an important direction for future examination.

Finally, although we conducted bias analyses for selection bias, the extent of possible selection bias is unknown and could have a large influence on study results.

To our knowledge, our study is the first to look at the association of neighborhood SES with quality of CKD care received not restricted to ESKD. Major strengths of our study include EHR data from routine clinical care, availability of geographic data, and using small geographic areas to determine neighborhood SES.

In conclusion, overall quality of care in patients with CKD can be greatly improved. EHRs can be leveraged to assess adherence to evidence-based screening and treatment guidelines in the care of patients with CKD and can target providers, the health care system, and patients.^43^^,^^44^ Despite previous evidence that neighborhood SES influences quality of health care for conditions such as CVD and diabetes,^10^, ^11^, ^12^, ^13^ we found no association of neighborhood SES with overall quality of care in patients with CKD. Quality improvement initiatives focusing on prevention, screening, and improved management of patients with multiple comorbid conditions are needed.

## Supplementary Material

Supplementary File (PDF) Item S1, Tables S1-S14
