# Supplementary material for: Neighborhood Socioeconomic Status and Quality of Kidney Care: Data From Electronic Health Records
Source: Kidney Med. 2021 Apr 19;3(4):515–527.e1. doi: 10.1016/j.xkme.2021.02.008 (PMC8350826; doi:10.1016/j.xkme.2021.02.008)
Supplement: Supplementary File (PDF) — Item S1, Tables S1-S14 [file mmc1.pdf]

Item S1. Definitions

Fairview Health System:
Fairview is a nonprofit organization, here for every health care need and every Minnesotan. They provide a network of more than 5,000 doctors and providers at primary care and specialty clinics across the state. This includes 12 hospitals and medical centers, 56 primary care clinics and 36 pharmacies.
https://www.fairview.org/

Defining patient moving:
We have geocoded addresses of all patients in our cohort. A patient was considered to have stayed in the same residence unless a new address was documented during the cohort period July 1, 2017 and December 31, 2018. Addresses are regularly updated at clinic visits across the Fairview Health System.

Defining primary care physician visit:
It is a completed outpatient office visits to family practice, family practice-internal medicine, internal medicine, obstetrics/gynecology, gerontology or geriatrics clinic visit

Defining race:
We used electronic health record data. Race data is usually inputted at first visit to Fairview clinic by an administrator or nurse after asking the patient. A patients' race was considered black if at any time patient has identified as belonging to black race; of the remaining participants we identified sequentially "Asian", "American Indian or Alaska Native or Native Hawaiian or other", "white", and missing/not recorded race.

Only 0.6-1% of our cohort are American Indian or Alaska native or Native Hawaiian and 4-5% Asian and 3-4% with missing race. For all models we adjusted for race as a categorical variable of black vs. non-black.

Defining insurance:
Patients insurance status was defined as a 2 level categorical variable:
Private insurance [private insurance (if <65) and private supplemental insurance (if ≥ 65)] vs. non-private insurance [Medicaid (if <65) and Medicare (if ≥ 65)]. We had <1% of patients who were uninsured.

For patients $\geq$ 65 years they were considered to be on **Medicare** if coded as:
MEDICARE PT B ONLY
MEDICARE PT A
RR MEDICARE PT A ONLY
MEDICARE RAILROAD PART B ONLY
MEDICARE
MEDICARE HOMECARE & HOSPICE
ACO MEDICARE
UCARE MEDICARE [Medicare advantage plans]
MEDICARE ADVANTAGE
HEALTHPARTNERS MEDICARE ADVANTAGE
HUMANA MEDICARE ADVANTAGE
UNITED HEALTHCARE MEDICARE ADVANTAGE
ATENA MEDICARE ADVANTAGE
OSF MEDICARE ADVANTAGE
BCBS MEDICARE ADVANTAGE
i.e. Medicare Plans A, B and C

Body Mass Index (BMI) missingness:
For the cohort used to assess angiotensin medication prescription BMI was missing in 933/16776 (5.6%) patients. For the cohort used to assess chronic kidney disease identification and urine albumin to creatinine ratio measurement, BMI was missing in 1,838/25,097 (7.3%) of patients.

Chart Review Details:
The manual review of charts was done by one person. Only patient ID's were given to the adjudicator. The adjudicator then obtained all relevant clinical and lab data. Once data collection was completed it was compared to data obtained directly from the EHR and used for the analysis. There was no knowledge of EHR coding by the adjudicator.

Table S1. Quantitative selection bias analysis for association of tract level socioeconomic status with angiotensin prescription compliance by providers

	Crude Poisson regression PR	Selection bias adjusted crude PR
**Median value of owner-occupied housing units is the measure of SES**		
High SES tract	1.0	1.0
Low SES tract (A)	0.98	0.91
Low SES tract (B)		0.69
Low SES tract (C)		0.77
Low SES tract (D)		1.13
Low SES tract (E)		1.11
**%>25 years with a Bachelor's degree or more**		
High SES tract	1.0	1.0
Low SES tract (A)	1.0	1.01
Low SES tract (B)		0.66
Low SES tract (C)		0.84
Low SES tract (D)		1.20
Low SES tract (E)		1.17
**Median household income and insurance status**		
High SES tract	1.0	1.0
Low SES tract (A)	0.96	0.88
Low SES tract (B)		0.66
Low SES tract (C)		0.74
Low SES tract (D)		1.16
Low SES tract (E)		1.08

PR: prevalence ratio

(A), (B), (C), (D), and (E) refer to different selection scenarios. Details are shown below.

Angiotensin prescription compliant +: patient included in our cohort given that they have angiotensin medications prescribed per guidelines
Angiotensin prescription compliant -: patient included in our cohort given that angiotensin medications were NOT prescribed per guidelines
Low SES +: patient living in low SES tract (first quartile of each of the tract measures)
Low SES -: patient living in a high SES tract (fourth quartile of each of the tract measures)

<u>Assumptions for scenarios (A), (B), and (C):</u> $S_1 \leq S_2$, $S_1 > S_3$, $S_2 > S_3$ and $S_3 < S_4$

	S_1 (Angiotensin prescription compliant + & low SES+)	S_2 (Angiotensin prescription compliant + & low SES-)	S_3 (Angiotensin prescription compliant - & low SES+)	S_4 (Angiotensin prescription compliant - & low SES-)
**Scenario A**	0.5	0.9	0.2	0.4
**Scenario B**	0.4	0.7	0.1	0.3
**Scenario C**	0.6	0.6	0.3	0.5

Assumptions for scenarios (D) and (E): $S_1<S_3$; $S_3= S_4$; $S_2>S_1$ and $S_2>S_4$

	S_1 (Angiotensin prescription compliant + & low SES+)	S_2 (Angiotensin prescription compliant + & low SES-)	S_3 (Angiotensin prescription compliant - & low SES+)	S_4 (Angiotensin prescription compliant - & low SES-)
**Scenario D**	0.4	0.6	0.5	0.5
**Scenario E**	0.5	0.7	0.6	0.6

For example under scenario A:
For S_1=0.5, that means the probability of a patient being included in our cohort given that they have angiotensin medications prescribed per guidelines and living in a low SES tract (first quartile) is 50%.

For S_2=0.9, that means the probability of a patient being included in our cohort given that they have angiotensin medications prescribed per guidelines and living in a high SES tract (fourth quartile) is 90%. We are assuming that $S_1 \leq S_2$ i.e. high SES are more likely to be included in our cohort than low SES.

For S3 =0.2, that means the probability of a patient being included in our cohort given that angiotensin medications were NOT prescribed per guidelines and living in a low SES tract (first quartile) is 20%. We are assuming that $S_1 > S_3$ that is the probability of a patient included in our cohort living in a low SES tract receiving angiotensin medications prescribed per guidelines is higher than a patient included in our cohort living in a low SES tract NOT receiving angiotensin medications prescribed per guidelines. The assumption here is if the patient makes it to Fairview to see a provider and they are low SES, they are more likely to be sicker and have more comorbidities (such as hypertension) so the provider is likely to prescribe appropriate medications that not.

For S4=0.4, that means the probability of a patient being included in our cohort given that angiotensin medications were NOT prescribed per guidelines and living in a high SES tract (fourth quartile) is 40%. We are assuming $S_3<S_4$ i.e high SES are more likely to be included in cohort than low SES.

For scenario A, here are the raw data for the association of NSES (median value of owner occupied housing units) and ACEi/ARB prescription by provider:

	Observed Data		Missing Data		Corrected for Selection Proportions	
	low SES +	low SES -	low SES +	low SES -	low SES +	low SES -
Ang prescribed +	1022 (a)	4426 (b)	1022.0 (A_1)	491.8 (B_1)	2044.0 (A_0)	4917.8 (B_0)
Ang prescribed -	585 (c)	2399 (d)	2340.0 (C_1)	3598.5 (D_1)	2925.0 (C_0)	5997.5 (D_0)
Total	1607 (m)	6825 (n)	3362.0 (M_1)	4090.3 (N_1)	4969.0 (M_0)	10915.3 (N_0)

Observed PR=0.98 Missing data Stratum PR=2.53 **Adjusted PR:0.91**

This is repeated for each different scenario. The assumptions and the selection probabilities shown are subject values.

Reference: Lash TL FM, Fink AK. Applying Quantitative Bias Analysis to Epidemiologic Data. Springer. 2009.
This is the link to the selection bias spreadsheet used: https://drive.google.com/file/d/10yEuOM4uLZkb3Bv_T1FxIF7RzjQjjzMh/view

Table S2.A Quantitative selection bias analysis for association of tract level socioeconomic status with urine albumin to creatinine (UACR) measurement

	Crude Poisson regression PR	Selection bias adjusted crude PR
**Median value of owner-occupied housing units is the measure of SES**		
High SES tract	1.0	1.0
Low SES tract (A)	1.2	1.13
Low SES tract (B)		0.77
Low SES tract (C)		0.80
Low SES tract (D)		1.60
Low SES tract (E)		1.52
**%>25 years with a Bachelor's degree or more**		
High SES tract	1.0	1.0
Low SES tract (A)	1.3	1.28
Low SES tract (B)		0.88
Low SES tract (C)		0.91
Low SES tract (D)		1.78
Low SES tract (E)		1.7
**Median household income and insurance status**		
High SES tract	1.0	1.0
Low SES tract (A)	0.97	0.89
Low SES tract (B)		0.60
Low SES tract (C)		0.65
Low SES tract (D)		1.31
Low SES tract (E)		1.25

PR: prevalence ratio

(A), (B), (C), (D), and (E) refer to different selection scenarios. Details are shown below.

UACR measured +: patient included in our cohort given that UACR was measured
UACR measured -: patient included in our cohort given that UACR was NOT measured
Low SES +: patient living in low SES tract (first quartile of each of the tract measures)
Low SES -: patient living in a high SES tract (fourth quartile of each of the tract measures)

<u>**Assumptions for scenarios (A), (B), and (C):**</u> $S_1 \leq S_2$, $S_1 > S_3$, $S_2 > S_3$ and $S_3 < S_4$

	S_1 (UACR measured +& low SES+)	S_2 (UACR measured +& low SES-)	S_3 (UACR measured -& low SES+)	S_4 (UACR measured -& low SES-)
**Scenario A**	0.5	0.9	0.2	0.4
**Scenario B**	0.4	0.7	0.1	0.3
**Scenario C**	0.6	0.6	0.3	0.5

<u>Assumptions for scenarios (D) and (E):</u> $S_1 < S_3$; $S_3 = S_4$; $S_2 > S_1$ and $S_2 > S_4$

	S_1 (UACR measured +& low SES+)	S_2 (UACR measured +& low SES-)	S_3 (UACR measured -& low SES+)	S_4 (UACR measured -& low SES-)
**Scenario D**	0.4	0.6	0.5	0.5
**Scenario E**	0.5	0.7	0.6	0.6

<u>For example under scenario C:</u>
For S_1=0.6, that means the probability of a patient being included in our cohort given that UACR was measured and living in a low SES tract (first quartile) is 50%.

For S_2=0.6, that means the probability of a patient being included in our cohort given that UACR was measured and living in a high SES tract (fourth quartile) is 90%. We are assuming that $S_1 \leq S_2$ i.e. in this case high SES are equally likely to be included in our cohort than low SES among those who had UACR measured.

For S3 =0.3, that means the probability of a patient being included in our cohort given that UACR was NOT measured and living in a low SES tract (first quartile) is 20%. We are assuming that $S_1 > S_3$ that is the probability of a patient included in our cohort living in a low SES tract and having UACR measured is higher than a patient included in our cohort living in a low SES tract and did NOT have UACR measured. The assumption here is if the patient makes it to Fairview to see a provider and they are low SES, they are more likely to be sicker and have more comorbidities (such as hypertension) so the provider is likely to measure UACR than not.

For S4=0.5, that means the probability of a patient being included in our cohort given that UACR was NOT measured and living in a high SES tract (fourth quartile) is 40%. We are assuming $S_3 < S_4$ i.e high SES are more likely to be included in cohort than low SES.

For scenario C, here are the raw data for the association of NSES (median value of owner occupied housing units) and UACR measured:

Observed Data

	low SES +	low SES -
**UACR measured +**	692 (a)	2590 (b)
**UACR measured -**	1683 (c)	8021 (d)
**Total**	2375 (m)	10611 (n)

Missing Data

	low SES +	low SES -
**UACR measured +**	461.3 (A_1)	1726.7 (B_1)
**UACR measured -**	3927.0 (C_1)	8021.0 (D_1)
**Total**	4388.3 (M_1)	9747.7 (N_1)

Corrected for Selection Proportions

	low SES +	low SES -
**UACR measured +**	1153.3 (A_0)	4316.7 (B_0)
**UACR measured -**	5610.0 (C_0)	16042.0 (D_0)
**Total**	6763.3 (M_0)	20358.7 (N_0)

Observed PR=1.19 Missing data Stratum PR=0.59 **Adjusted PR:0.80**

This is repeated for each different scenario. The assumptions and the selection probabilities shown are subject values.
Reference: Lash TL FM, Fink AK. Applying Quantitative Bias Analysis to Epidemiologic Data. Springer. 2009.
This is the link to the selection bias spreadsheet used: https://drive.google.com/file/d/10yEuOM4uLZkb3Bv_T1FxIF7RzjQjjzMh/view

Table S2.B Quantitative selection bias analysis for association of tract level socioeconomic status with chronic kidney disease (CKD) identified

	Crude Poisson regression PR	Selection bias adjusted crude PR
**Median value of owner-occupied housing units is the measure of SES**		
High SES tract	1.0	1.0
Low SES tract (A)	1.2	1.15
Low SES tract (B)		0.85
Low SES tract (C)		0.90
Low SES tract (D)		1.38
Low SES tract (E)		1.34
**%>25 years with a Bachelor's degree or more**		
High SES tract	1.0	1.0
Low SES tract (A)	1.2	1.15
Low SES tract (B)		0.86
Low SES tract (C)		0.90
Low SES tract (D)		1.38
Low SES tract (E)		1.34
**Median household income and insurance status**		
High SES tract	1.0	1.0
Low SES tract (A)	1.1	1.03
Low SES tract (B)		0.76
Low SES tract (C)		0.82
Low SES tract (D)		1.28
Low SES tract (E)		1.24

PR: prevalence ratio

(A), (B), (C), (D), and (E) refer to different selection scenarios. Details are shown below.

CKD identified +: patient included in our cohort given that CKD was identified in the EHR
CKD identified -: patient included in our cohort given that CKD was NOT identified in the EHR
Low SES +: patient living in low SES tract (first quartile of each of the tract measures)
Low SES -: patient living in a high SES tract (fourth quartile of each of the tract measures)

Assumptions for scenarios (A), (B), and (C): $S_1 \leq S_2$, $S_1 > S_3$, $S_2 > S_3$ and $S_3 < S_4$

	S_1 (CKD identified + & low SES+)	S_2 (CKD identified + & low SES-)	S_3 (CKD identified - & low SES+)	S_4 (CKD identified - & low SES-)
**Scenario A**	0.5	0.9	0.2	0.4
**Scenario B**	0.4	0.7	0.1	0.3
**Scenario C**	0.6	0.6	0.3	0.5

<u>Assumptions for scenarios (D) and (E):</u> $S_1<S_3$; $S_3= S_4$; $S_2>S_1$ and $S_2>S_4$

	S_1 (CKD identified + & low SES+)	S_2 (CKD identified + & low SES-)	S_3 (CKD identified - & low SES+)	S_4 (CKD identified - & low SES-)
**Scenario D**	0.4	0.6	0.5	0.5
**Scenario E**	0.5	0.7	0.6	0.6

<u>For example under scenario E:</u>
For S_1=0.5, that means the probability of a patient being included in our cohort given that CKD was identified in the EHR and living in a low SES tract (first quartile) is 50%.

For S_2=0.7, that means the probability of a patient being included in our cohort given that CKD was identified in the EHR and living in a high SES tract (fourth quartile) is 90%. We are assuming that $S_1 < S_2$ i.e. in this case high SES are less likely to be included in our cohort.

For S3 =0.6, that means the probability of a patient being included in our cohort given that CKD was NOT identified in the EHR and living in a low SES tract (first quartile) is 60%. We are assuming that $S_1 < S_3$ that is the probability of a patient included in our cohort living in a low SES tract and having CKD identified in the EHR is lower than a patient included in our cohort living in a low SES tract and did NOT have CKD identified in the EHR.

For S4=0.6, that means the probability of a patient being included in our cohort given that CKD was NOT identified in the EHR and living in a high SES tract (fourth quartile) is 40%. We are assuming S_3=S_4 i.e high and low SES are equally likely to be included in cohort among those who did not have CKD identified in the EHR.

For scenario E, here are the raw data for the association of NSES (median value of owner occupied housing units) and CKD identified in the EHR:

	Observed Data: low SES +	Observed Data: low SES -	Missing Data: low SES +	Missing Data: low SES -	Corrected for Selection Proportions: low SES +	Corrected for Selection Proportions: low SES -
CKD identified +	1406 (a)	5448 (b)	1406.0 (A_1)	2334.9 (B_1)	2812.0 (A_0)	7782.9 (B_0)
CKD identified -	969 (c)	5163 (d)	646.0 (C_1)	3442.0 (D_1)	1615.0 (C_0)	8605.0 (D_0)
Total	2375 (m)	10611 (n)	2052.0 (M_1)	5776.9 (N_1)	4427.0 (M_0)	16387.9 (N_0)

Observed PR=1.15 Missing data Stratum PR=1.7 **Adjusted PR:1.34**

This is repeated for each different scenario. The assumptions and the selection probabilities shown are subject values.

Reference: Lash TL FM, Fink AK. Applying Quantitative Bias Analysis to Epidemiologic Data. Springer. 2009.
This is the link to the selection bias spreadsheet used: https://drive.google.com/file/d/10yEuOM4uLZkb3Bv_T1FxIF7RzjQjjzMh/view

Table S3. Characteristics of nonpregnant adults with hypertension and chronic kidney disease* and by angiotensin prescription compliance by providers**

	**Overall** N=16,776	**Angiotensin medication prescription compliant** N=10,885	**Non-compliant with angiotensin medication prescription** N=5,891
**eGFR (ml/min/1.73m^2), mean(SD)**	47.7 ± 15.3	49.4 ± 15.3	44.5 ± 14.7
**Individual level demographic characteristics**			
Age, mean (SD)	72.8 ± 13.4	72.6 ± 12.9	73.2 ± 14.3
Male, n(%)	7,108 (42%)	4,719 (43%)	2,389 (41%)
Black, n(%)	885 (5%)	583 (5%)	302 (5%)
**Individual social characteristics (n%)**			
Ever smokers, n(%)	8,485 (51%)	5,489 (50%)	2,996 (51%)
Medicaid (among adults <65yrs), n(%)	287 (7%)	163 (6%)	124 (8%)
Medicare (among adults ≥65yrs), n(%)	2,767 (22%)	1,882 (23%)	885 (20%)
**Medical History**			
Diabetes, n(%)	6,149 (37%)	4,423 (41%)	1,726 (29%)
Obese (BMI≥ 30 kg/m^2), n(%)	7,088 (45%)	5,014 (48%)	2,074 (38%)
Cardiovascular disease, n(%)	7,277 (43%)	4,606 (42%)	2,671 (45%)
Stroke, n(%)	1,906 (11%)	1,222 (11%)	684 (12%)
Hyperlipidemia, n(%)	13,122 (78%)	9,047 (83%)	4,075 (69%)
Cancer, n(%)	2,651 (16%)	1,599 (15%)	1,052 (18%)
**Median value of owner occupied housing units**			
Q1: < $165,200	1,607 (10%)	1,022 (9%)	585 (10%)
Q2: $165,200 - $188,100	2,196 (13%)	1,482 (14%)	714 (12%)
Q3: $188,100 - $231,300	6,144 (37%)	3,952 (36%)	2,192 (37%)
Q4: ≥ $231,300	6,825 (41%)	4,426 (41%)	2,399 (41%)
**Percent of residents > 25 years with a Bachelor's degree or more**			
Q1: < 20.4%	1,945 (12%)	1,295 (12%)	650 (11%)
Q2: 20.4% - 34.1%	4,560 (27%)	3,011 (28%)	1,549 (26%)
Q3: 34.1% - 48.1%	5,668 (34%)	3,638 (33%)	2,030 (35%)
Q4: ≥ 48.1%	4,600 (27%)	2,939 (27%)	1,661 (28%)
**Median household income**			
Q1: <$35,935	1,807 (11%)	1,137 (11%)	670 (11%)
Q2: $35,935 - $47,379	2,334 (14%)	1,521 (14%)	813 (14%)
Q3: $47,379 - $62,343	3,956 (24%)	2,532 (23%)	1,424 (24%)
Q4: ≥ $62,343	8,664 (52%)	5,688 (52%)	2,976 (51%)

*estimated glomerular filtration rate <60/mL/min per 1.73m^2 from 7/1/2017 to 12/31/2018

** Adults (nonpregnant patients) with hypertension and chronic kidney disease (stage 3 or higher, or stage 1 or 2 with UACR >300mg/day) should be taking angiotensin converting enzyme inhibitor (ACEi) and angiotensin receptor blocker (ARB)

ACEI/ARB prescription adherence: yes if recommended ACEi/ARB prescribed

CKD: chronic kidney disease; cardiovascular disease includes congestive heart failure, acute myocardial infarction, ischemic heart disease, and peripheral vascular disease

Table S4. Overall characteristics of adults with chronic kidney disease* and of those who had UACR measured vs. not**

	**Overall** N=25,097	**UACR measured** N= 6,863	**UACR not measured** N= 18,234
**eGFR (ml/min/1.73m^2), mean(SD)**	46.9 ± 12.31	46.0 ± 11.3	47.2 ± 12.7
**Individual level demographic characteristics**			
Age, mean (SD)	70.9 ± 14.1	71.9 ± 12.3	70.5 ± 14.7
Male, n(%)	10,323 (41%)	2,999 (44%)	7,324 (40%)
Black, n(%)	1,130 (5%)	368 (6%)	744 (4%)
**Individual social characteristics (n%)**			
Ever smokers, n(%)	12,254 (49%)	3,510 (51%)	8,744 (48%)
Medicaid (among adults <65yrs), n(%)	539 (7%)	115 (6%)	424 (7%)
Medicare (among adults ≥65yrs), n(%)	3,707 (21%)	1,258 (25%)	2,449 (20%)
**Medical History**			
Hypertension, n(%)	19,627 (78%)	6,389 (93%)	13,238 (73%)
Diabetes, n(%)	7,763 (31%)	4,271 (62%)	3,492 (19%)
Obese (BMI≥ 30 kg/m^2), n(%)	9,674 (42%)	3,424 (51%)	6,250 (38%)
Cardiovascular disease, n(%)	9,527 (38%)	2,813 (41%)	6,714 (37%)
Stroke, n(%)	2,512 (10%)	766 (11%)	1,746 (10%)
Hyperlipidemia, n(%)	17,334 (69%)	5,968 (87%)	11,366 (62%)
Cancer, n(%)	3,772 (15%)	1,018 (15%)	2,754 (15%)
**Median value of owner occupied housing units**			
Q1: < $165,200	2,375 (10%)	692 (10%)	1,683 (9%)
Q2: $165,200 - $188,100	3,164 (13%)	1,013 (15%)	2,151 (12%)
Q3: $188,100 - $231,300	8,940 (36%)	2,567 (37%)	6,373 (35%)
Q4: ≥ $231,300	10,611 (42%)	2,590 (38%)	8,021 (44%)
**Percent of residents > 25 years with a Bachelor's degree or more**			
Q1: < 20.4%	2,795 (11%)	865 (13%)	1,930 (11%)
Q2: 20.4% - 34.1%	6,673 (27%)	1,983 (29%)	4,690 (26%)
Q3: 34.1% - 48.1%	8,450 (34%)	2,347 (34%)	6,103 (34%)
Q4: ≥ 48.1%	7,173 (29%)	1,667 (24%)	5,506 (30%)
**Median household income**			
Q1: <$35,935	2,723 (11%)	733 (11%)	1,990 (11%)
Q2: $35,935 - $47,379	3,391 (14%)	928 (14%)	2,463 (14%)
Q3: $47,379 - $62,343	5,857 (23%)	1,608 (23%)	4,249 (23%)
Q4: ≥ $62,343	13,104 (52%)	3,590 (52%)	9,514 (52%)

*estimated glomerular filtration rate <60/mL/min per 1.73m^2 from 7/1/2017 to 12/31/2018
**UACR measured: UACR measured in patients with CKD (eGFR <60 ml/min/1.73m^2).
CKD: chronic kidney disease; cardiovascular disease includes congestive heart failure, acute myocardial infarction, ischemic heart disease, and peripheral vascular disease

Table S5. Characteristics of adults with chronic kidney disease* who had CKD identified in the electronic health records vs. not**

	**CKD identified** N=13,811	**CKD not identified** N=11,286
**eGFR (ml/min/1.73m^2), mean(SD)**	41.9 ± 13.6	52.9 ± 6.7
**UACR (mg/day), mean(SD)**[1]	371.2 ± 1033.1	124.2 ± 586.9
**Albuminuria (>300mg/day), n(%)**[1]	1,021 (20%)	108 (7%)
**Individual level demographic characteristics**		
Age, mean (SD)	72.5 ± 14.1	68.9 ± 13.9
Male, n(%)	6,405 (46%)	3,918 (35%)
Black, n(%)	906 (7%)	224 (2%)
**Individual social characteristics (n%)**		
Ever smokers, n(%)	7,158 (52%)	5,096 (45%)
Medicaid (among adults <65yrs), n(%)	361 (10%)	178 (4%)
Medicare (among adults ≥65yrs), n(%)	2,228 (22%)	1,479 (15%)
**Medical History**		
Hypertension, n(%)	12,561 (91%)	7,066 (63%)
Diabetes, n(%)	5,808 (42%)	1,955 (17%)
Obese (BMI≥ 30 kg/m^2), n(%)	5,521 (42%)	4,153 (41%)
Cardiovascular disease, n(%)	6,742 (49%)	2,785 (25%)
Stroke, n(%)	1,789 (13%)	723 (6%)
Hyperlipidemia, n(%)	10,841 (79%)	6,493 (58%)
Cancer, n(%)	2,330 (17%)	1,442 (13%)
**Median value of owner occupied housing units**		
Q1: < $165,200	1,406 (10%)	969 (9%)
Q2: $165,200 - $188,100	1,863 (14%)	1,301 (12%)
Q3: $188,100 - $231,300	5,091 (37%)	3,849 (34%)
Q4: ≥ $231,300	5,448 (40%)	5,163 (46%)
**Percent of residents > 25 years with a Bachelor's degree or more**		
Q1: < 20.4%	1,652 (12%)	1,143 (10%)
Q2: 20.4% - 34.1%	3,820 (28%)	2,853 (25%)
Q3: 34.1% - 48.1%	4,671 (34%)	3,779 (34%)
Q4: ≥ 48.1%	3,666 (27%)	3,507 (31%)
**Median household income**		
Q1: <$35,935	1,542 (11%)	1,181 (11%)
Q2: $35,935 - $47,379	1,978 (14%)	1,413 (13%)
Q3: $47,379 - $62,343	3,323 (24%)	2,535 (23%)
Q4: ≥ $62,343	6,954 (50%)	6,150 (55%)

*estimated glomerular filtration rate <60/mL/min per 1.73m^2 from 7/1/2017 to 12/31/2018

**CKD identification: CKD documented in EHR, using ICD9/10 codes, among patients with CKD (≤60 ml/min/1.73m^2)

CKD: chronic kidney disease; cardiovascular disease includes congestive heart failure, acute myocardial infarction, ischemic heart disease, and peripheral vascular disease

UACR measured was missing in 8,600/13,811 of patients who had CKD identified in the electronic health records (defined as having at least two International Classification of Disease (ICD) 9/10 codes for CKD, ESKD, dialysis, or kidney transplant documented before and up until 90 days after the date of the index creatinine among patients with lab-based CKD) and UACR measured was missing in 9,634/11,286 of patients who did not have CKD identified (based on eGFR). Results shown are for 5,211 patients with CKD identified and 1,652 of patients with CKD not identified.

Table S6.A Kidney care measures and neighborhood socioeconomic status

	**Overall**		
	**ACEI/ARB prescription compliance**	**Urine albumin to creatinine ratio (UACR) measured**	**CKD identified in the EHR**
**Median value of owner occupied housing units, n/N (%)**			
Q1: < $165,200	1,022/1,607 (64%)	692/2,375 (29%)	1,406/2,375 (59%)
Q2: $165,200 - $188,100	1,482/2,196 (67%)	1,013/3,164 (32%)	1,863/3,164 (59%)
Q3: $188,100 - $231,300	3,952/6,144 (64%)	2,567/8,940 (29%)	5,091/8,940 (57%)
Q4: ≥ $231,300	4,426/6,825 (65%)	2,590/10,611 (24%)	5,448/10,611 (51%)
**Percent of Residents > 25 years with a Bachelor’s degree or more, n/N (%)**			
Q1: < 20.4%	1,295/1,945 (67%)	865/2,795 (31%)	1,652/2,795 (59%)
Q2: 20.4% - 34.1%	3,011/4,560 (66%)	1,983/6,673 (30%)	3,820/6,673 (57%)
Q3: 34.1% - 48.1%	3,638/5,668 (64%)	2,347/8,450 (28%)	4,671/8,450 (55%)
Q4: ≥ 48.1%	2,939/4,600 (64%)	1,667/7,173 (23%)	3,666/7,173 (51%)
**Median household income, n/N (%)**			
Q1: <$35,935	1,137/1,807 (63%)	733/2,723 (27%)	1,542/2,723 (56%)
Q2: $35,935 - $47,379	1,521/2,334 (65%)	928/3,391 (27%)	1,978/3,391 (58%)
Q3: $47,379 - $62,343	2,532/3,956 (64%)	1,608/5,857 (27%)	3,323/5,857 (57%)
Q4: ≥ $62,343	5,688/8,664 (66%)	3,590/13,104 (27%)	6,954/13,104 (53%)

For angiotensin medication prescription compliance:
n/N shown is = #of patients who are angiotensin medication prescription compliant for quartile x of neighborhood socioeconomic status measure (z)/ Total # of patients in quartile x of neighborhood socioeconomic status measure (z)
x is 1st quartile, 2nd quartile, 3rd quartile, or 4th quartile
z is median value of owner occupied housing units, percent of residents >25 years with a Bachelor’s degree or more and median household income
For UACR measured:
n/N shown is = #of patients in which UACR was measured for quartile x of neighborhood socioeconomic status measure (z)/ Total # of patients in quartile x of neighborhood socioeconomic status measure (z)
x is 1st quartile, 2nd quartile, 3rd quartile, or 4th quartile
z is median value of owner occupied housing units, percent of residents >25 years with a Bachelor’s degree or more and median household income
For CKD identification in the EHR:
n/N shown is = #of patients in which CKD was identified for quartile x of neighborhood socioeconomic status measure (z)/ Total # of patients in quartile x of neighborhood socioeconomic status measure (z)
x is 1st quartile, 2nd quartile, 3rd quartile, or 4th quartile
z is median value of owner occupied housing units, percent of residents >25 years with a Bachelor’s degree or more and median household income

Table S6.B Kidney care measures and neighborhood socioeconomic status for blacks and non-blacks

	**Blacks**		
	**ACEI/ARB prescription compliance**	**Urine albumin to creatinine ratio (UACR) measured**	**CKD identified in the EHR**
**Median value of owner occupied housing units, n/N(%)**			
Q1: < $165,200	148/229 (65%)	95/292 (32%)	224/292 (77%)
Q2: $165,200 - $188,100	103/158 (65%)	72/210 (34%)	168/210 (80%)
Q3: $188,100 - $231,300	207/311 (67%)	136/383 (36%)	320/383 (84%)
Q4: ≥ $231,300	125/187 (67%)	83/245 (34%)	194/245 (79%)
**Percent of Residents > 25 years with a Bachelor's degree or more, n/N(%)**			
Q1: < 20.4%	128/209 (61%)	83/254 (33%)	198/254 (78%)
Q2: 20.4% - 34.1%	182/276 (66%)	124/364 (34%)	297/364 (82%)
Q3: 34.1% - 48.1%	177/247 (72%)	118/323 (37%)	261/323 (81%)
Q4: ≥ 48.1%	96/153 (63%)	61/189 (32%)	150/189 (79%)
**Median household income, n(%)**			
Q1: <$35,935	130/205 (63%)	89/260 (34%)	221/260 (78%)
Q2: $35,935 - $47,379	91/134 (68%)	65/191 (34%)	205/191 (81%)
Q3: $47,379 - $62,343	162/241 (67%)	90/303 (30%)	235/303 (81%)
Q4: ≥ $62,343	199/303 (66%)	142/374 (38%)	245/374 (80%)
	**Non-Blacks**		
**Median value of owner occupied housing units, n/N(%)**			
Q1: < $165,200	874/1,378 (63%)	597/2,083 (29%)	1,182/2,083 (57%)
Q2: $165,200 - $188,100	1,379/2,038 (68%)	941/2,954 (32%)	1,695/2,954 (57%)
Q3: $188,100 - $231,300	3,745/5,833 (64%)	2,431/8,557 (28%)	4,771/8,557 (56%)
Q4: ≥ $231,300	4,301/6,638 (65%)	2,507/10,366 (24%)	5,254/10,366 (51%)
**Percent of Residents > 25 years with a Bachelor's degree or more, n/N(%)**			
Q1: < 20.4%	1,167/1,736 (67%)	782/2,541 (31%)	1,454/2,541 (57%)
Q2: 20.4% - 34.1%	2,829/4,284 (66%)	1,859/6,309 (29%)	3,523/6,309 (56%)
Q3: 34.1% - 48.1%	3,461/5,421 (64%)	2,229/8,127 (27%)	4,410/8,127 (54%)
Q4: ≥ 48.1%	2,843/4,447 (64%)	1,606/6,984 (23%)	3,516/6,984 (50%)
**Median household income, n/N(%)**			
Q1: <$35,935	1,007/1,602 (63%)	644/2,463 (26%)	1,330/2,463 (54%)
Q2: $35,935 - $47,379	1,430/2,200 (65%)	863/3,200 (27%)	1,826/3,200 (57%)
Q3: $47,379 - $62,343	2,370/3,715 (64%)	1,518/5,554 (27%)	3,074/5,554 (55%)
Q4: ≥ $62,343	5,489/8,361 (66%)	3,448/12,730 (27%)	6,662/12,730 (52%)

Values are calculated for Blacks and Non-Blacks separately

For angiotensin medication prescription compliance:

n/N shown is = #of patients who are angiotensin medication prescription compliant for quartile x of neighborhood socioeconomic status measure (z)/ Total # of patients in quartile x of neighborhood socioeconomic status measure (z)

x is 1st quartile, 2nd quartile, 3rd quartile, or 4th quartile

z is median value of owner occupied housing units, percent of residents >25 years with a Bachelor's degree or more and median household income

For UACR measured:

n/N shown is = #of patients in which UACR was measured for quartile x of neighborhood socioeconomic status measure (z)/ Total # of patients in quartile x of neighborhood socioeconomic status measure (z)

x is 1st quartile, 2nd quartile, 3rd quartile, or 4th quartile

z is median value of owner occupied housing units, percent of residents >25 years with a Bachelor's degree or more and median household income

<u>For CKD identification in the EHR</u>:

n/N shown is = #of patients in which CKD was identified for quartile x of neighborhood socioeconomic status measure (z)/ Total # of patients in quartile x of neighborhood socioeconomic status measure (z)

x is 1st quartile, 2nd quartile, 3rd quartile, or 4th quartile

z is median value of owner occupied housing units, percent of residents >25 years with a Bachelor's degree or more and median household income

Table S7.A Multilevel regression model for the association of tract level socioeconomic status with urine albumin to creatinine measurement (CKD defined as eGFR<45 ml/min/1.73m^2)*

	Model 1 (PR, 95% CI)	Model 2 (PR, 95% CI)	Model 3 (PR, 95% CI)
**Median *value of owner-occupied housing units is the measure of SES***			
High SES tract – 4th Q (n=3,200)	1.00	1.00	1.00
3rd Q (n=2,994)	1.13 [1.03, 1.24]	1.09 [1.00, 1.20]	1.09 [1.00, 1.19]
2nd Q (n=1,145)	1.20 [1.07, 1.35]	1.15 [1.04, 1.28]	1.11 [1.01, 1.22]
Low SES tract – 1st Q (n=926)	1.11 [0.96, 1.29]	1.08 [0.94, 1.26]	1.06 [0.93, 1.21]
***Percent of residents >25 years with* a Bachelor's degree or more**			
High SES tract – 4th Q (n=2,132)	1.00	1.00	1.00
3rd Q (n=2,719)	1.18 [1.06, 1.32]	1.14 [1.02, 1.27]	1.11 [1.00, 1.22]
2nd Q (n=2,361)	1.17 [1.05, 1.30]	1.13 [1.01, 1.25]	1.09 [0.99, 1.21]
Low SES tract – 1st Q (n=1,053)	1.23 [1.07, 1.41]	1.18 [1.04, 1.35]	1.12 [1.00, 1.26]
Median household income			
High SES tract – 4th Q (n=3,985)	1.00	1.00	1.00
3rd Q (n=2,033)	0.95 [0.87, 1.07]	0.95 [0.86, 1.04]	0.98 [0.90, 1.07]
2nd Q (n=1,273)	0.93 [0.82, 1.06]	0.93 [0.82, 1.05]	0.93 [0.84, 1.04]
Low SES tract – 1st Q (n=974)	0.93 [0.81, 1.07]	0.95 [0.83, 1.09]	0.98 [0.87, 1.10]

*UACR measured: UACR measured in patients with CKD (**eGFR <45 ml/min/1.73m^2**). N total = 8,265 patients

SES: socioeconomic status, PR: prevalence ratio

Median value of owner-occupied housing units: high SES (4th quartile[Q]):≥$231,300, 3rd Q: $188,100-$231,300, 2nd Q: $165,200-$188,100, low SES(1st Q): <$165,200; %>25 years with a Bachelor's degree or more: high SES (4th quartile[Q]):≥48.1%, 3rd Q: 34.1%-48.1%, 2nd Q: 20.4%-34.1%, low SES(1st Q): <20.4%; Median household income: high SES (4th quartile[Q]):≥$62,343, 3rd Q: $47,379 - $62,343, 2nd Q: $35,935 - $47,379, low SES(1st Q): <$35,935

Model 1: crude
Model 2: age, sex, race, obesity, smoking, insurance status
Model 3: model 2 + history of cardiovascular disease, stroke, cancer, hyperlipidemia, diabetes, hypertension, index eGFR

Table S7.B Multilevel regression model for the association of tract level socioeconomic status with chronic kidney disease (CKD) identification (CKD defined as eGFR<45 ml/min/1.73m^2)*

	Model 1 (PR, 95% CI)	Model 2 (PR, 95% CI)	Model 3 (PR, 95% CI)
**Median *value of owner-occupied housing units is the measure of SES***			
High SES tract – 4th Q (n=3,200)	1.00	1.00	1.00
3rd Q (n=2,994)	1.03 [1.01, 1.06]	1.03 [1.01, 1.05]	1.01 [1.00, 1.03]
2nd Q (n=1,145)	1.03 [1.00, 1.06]	1.02 [0.98, 1.05]	1.01 [0.98, 1.03]
Low SES tract – 1st Q (n=926)	1.04 [1.01, 1.07]	1.03 [1.00, 1.06]	1.01 [0.98, 1.04]
***Percent of residents >25 years with* a Bachelor's degree or more**			
High SES tract – 4th Q (n=2,132)	1.00	1.00	1.00
3rd Q (n=2,719)	1.02 [0.99, 1.05]	1.02 [0.99, 1.04]	1.00 [0.98, 1.03]
2nd Q (n=2,361)	1.04 [1.02, 1.07]	1.03 [1.01, 1.06]	1.01 [0.99, 1.04]
Low SES tract – 1st Q (n=1,053)	1.04 [1.00, 1.07]	1.03 [1.00, 1.07]	1.02 [0.99, 1.05]
Median household income			
High SES tract – 4th Q (n=3,985)	1.00	1.00	1.00
3rd Q (n=2,033)	1.00 [0.98, 1.03]	1.00 [0.97, 1.02]	0.99 [0.97, 1.02]
2nd Q (n=1,273)	1.02 [0.99, 1.05]	1.01 [0.99, 1.05]	1.01 [0.99, 1.04]
Low SES tract – 1st Q (n=974)	0.99 [0.96, 1.02]	0.99 [0.96, 1.01]	0.99 [0.97, 1.02]

* CKD identified in EHR was defined as: number of patients who have CKD (**eGFR <45ml/min/1.73m^2**) documented by ICD9/10 codes in EHR. N total = 8,265 patients
SES: socioeconomic status, PR: prevalence ratio
Median value of owner-occupied housing units: high SES (4th quartile[Q]):≥$231,300, 3rd Q: $188,100-$231,300, 2nd Q: $165,200-$188,100, low SES(1st Q): <$165,200; %>25 years with a Bachelor's degree or more: high SES (4th quartile[Q]):≥48.1%, 3rd Q: 34.1%-48.1%, 2nd Q: 20.4%-34.1%, low SES(1st Q): <20.4%; Median household income: high SES (4th quartile[Q]):≥$62,343, 3rd Q: $47,379 - $62,343, 2nd Q: $35,935 - $47,379, low SES(1st Q): <$35,935

Model 1: crude
Model 2: age, sex, race, obesity, smoking, insurance status
Model 3: model 2 + history of cardiovascular disease, stroke, cancer, hyperlipidemia, diabetes, hypertension, index eGFR

Table S8.A Multilevel regression model for the association of tract level socioeconomic status with angiotensin medication prescription compliance* **(36 months - 12/31/2015 – 12/31/2018)**

	Model 1 (PR, 95% CI)	Model 2 (PR, 95% CI)	Model 3 (PR, 95% CI)
**Median *value of owner-occupied housing units is the measure of SES***			
High SES tract – 4th Q (n=8,314)	1.00	1.00	1.00
3rd Q (n=7,542)	0.99 [0.97, 1.03]	0.99 [0.97, 1.03]	0.99 [0.96, 1.02]
2nd Q (n=2,680)	1.05 [1.01, 1.10]	1.03 [0.99, 1.07]	1.02 [0.98, 1.06]
Low SES tract – 1st Q (n=2,087)	0.99 [0.93, 1.07]	0.98 [0.92, 1.04]	0.97 [0.92, 1.03]
***Percent of residents >25 years with* a Bachelor's degree or more**			
High SES tract (n=5,654) – 4th Q	1.00	1.00	1.00
3rd Q (n=6,938)	1.01 [0.97, 1.05]	1.01 [0.97, 1.07]	1.00 [0.97, 1.04]
2nd Q (n=5,582)	1.04 [0.99, 1.08]	1.03 [0.99, 1.07]	1.01 [0.98, 1.05]
Low SES tract – 1st Q(n=2,449)	1.06 [1.01, 1.10]	1.01 [0.98, 1.07]	1.01 [0.97, 1.05]
Median household income			
High SES tract – 4th Q (n=10,527)	1.00	1.00	1.00
3rd Q (n=4,895)	0.96 [0.93, 1.00]	0.99 [0.95, 1.02]	0.99 [0.95, 1.02]
2nd Q (n=2,931)	0.98 [0.94, 1.03]	1.00 [0.97, 1.05]	1.00 [0.96, 1.04]
Low SES tract – 1st Q (n=2,720)	0.96 [0.92, 1.00]	0.97 [0.93, 1.00]	0.97 [0.93, 1.01]

* Adults (nonpregnant patients) with hypertension and chronic kidney disease (stage 3 or higher, or stage 1 or 2 with UACR >300mg/day) should be taking angiotensin converting enzyme inhibitor (ACEI) and angiotensin receptor blocker (ARB)
ACEI/ARB prescription compliance: yes if recommended ACEI/ARB prescribed
Total cohort N= 20,623
SES: socioeconomic status, PR: prevalence ratio
Median value of owner-occupied housing units: high SES (4th quartile[Q]):≥$231,300, 3rd Q: $188,100-$231,300, 2nd Q: $165,200-$188,100, low SES(1st Q): <$165,200; %>25 years with a Bachelor's degree or more: high SES (4th quartile[Q]):≥48.1%, 3rd Q: 34.1%-48.1%, 2nd Q: 20.4%-34.1%, low SES(1st Q): <20.4%; Median household income: high SES (4th quartile[Q]):≥$62,343, 3rd Q: $47,379 - $62,343, 2nd Q: $35,935 - $47,379, low SES($1^{st}$ Q): <$35,935

Model 1: crude
Model 2: age, sex, race, obesity, smoking, insurance status
Model 3: model 2 + history of cardiovascular disease, stroke, cancer, hyperlipidemia, diabetes

Table S8.B Multilevel regression model for the association of tract level socioeconomic status with urine albumin to creatinine measurement **(36 months - 12/31/2015 – 12/31/2018)**

	Model 1 (PR, 95% CI)	Model 2 (PR, 95% CI)	Model 3 (PR, 95% CI)
**Median *value of owner-occupied housing units is the measure of SES***			
High SES tract – 4th Q (n=13,304)	1.00	1.00	1.00
3rd Q (n=11,204)	1.18 [1.11, 1.26]	1.13 [1.06, 1.20]	1.05 [0.99, 1.12]
2nd Q (n=3,939)	1.33 [1.22, 1.45]	1.23 [1.14, 1.33]	1.10 [1.03, 1.18]
Low SES tract – 1st Q (n=3,108)	1.21 [1.09, 1.33]	1.17 [1.63, 1.29]	1.03 [0.95, 1.12]
***Percent of residents >25 years with* a Bachelor's degree or more**			
High SES tract – 4th Q (n=9,027)	1.00	1.00	1.00
3rd Q (n=10,602)	1.17 [1.09, 1.26]	1.12 [1.04, 1.21]	1.06 [0.99, 1.13]
2nd Q (n=8,352)	1.28 [1.19, 1.38]	1.19 [1.11, 1.27]	1.06 [1.00, 1.13]
Low SES tract – 1st Q (n=3,574)	1.35 [1.23, 1.47]	1.25 [1.15, 1.36]	1.07 [0.99, 1.16]
Median household income			
High SES tract – 4th Q (n=16,361)	1.00	1.00	1.00
3rd Q (n=7,377)	1.00 [0.94, 1.07]	0.99 [0.94, 1.06]	0.97 [0.92, 1.03]
2nd Q (n=4,331)	0.97 [0.88, 1.06]	0.98 [0.90, 1.07]	0.94 [0.87, 1.01]
Low SES tract – 1st Q (n=3,486)	0.96 [0.87, 1.07]	0.99 [0.90, 1.09]	0.99 [0.89, 1.09]

*UACR measured: UACR measured in patients with CKD (eGFR <60 ml/min/1.73m^2).
Total cohort N= 31,555
SES: socioeconomic status, PR: prevalence ratio
Median value of owner-occupied housing units: high SES (4th quartile[Q]):≥$231,300, 3rd Q: $188,100-$231,300, 2nd Q: $165,200-$188,100, low SES(1st Q): <$165,200; %>25 years with a Bachelor's degree or more: high SES (4th quartile[Q]):≥48.1%, 3rd Q: 34.1%-48.1%, 2nd Q: 20.4%-34.1%, low SES(1st Q): <20.4%; Median household income: high SES (4th quartile[Q]):≥$62,343, 3rd Q: $47,379 - $62,343, 2nd Q: $35,935 - $47,379, low SES(1st Q): <$35,935

Model 1: crude
Model 2: age, sex, race, obesity, smoking, insurance status
Model 3: model 2 + history of cardiovascular disease, stroke, cancer, hyperlipidemia, diabetes, hypertension, index eGFR

Table S8.C Multilevel regression model for the association of tract level socioeconomic status with chronic kidney disease (CKD) identification **(36 months - 12/31/2015 – 12/31/2018)**

	Model 1 (PR, 95% CI)	Model 2 (PR, 95% CI)	Model 3 (PR, 95% CI)
**Median *value of owner-occupied housing units is the measure of SES***			
High SES tract – 4th Q (n=13,304)	1.00	1.00	1.00
3rd Q (n=11,204)	1.11 [1.08, 1.15]	1.08 [1.05, 1.11]	1.03 [1.09, 1.06]
2nd Q (n=3,939)	1.15 [1.09, 1.21]	1.11 [1.06, 1.17]	1.04 [0.99, 1.09]
Low SES tract – 1st Q (n=3,108)	1.15 [1.11, 1.20]	1.11 [1.07, 1.16]	1.02 [0.99, 1.06]
***Percent of residents >25 years with* a Bachelor's degree or more**			
High SES tract – 4th Q (n=9,027)	1.00	1.00	1.00
3rd Q (n=10,602)	1.08 [1.05, 1.12]	1.06 [1.03, 1.10]	1.02 [0.99, 1.05]
2nd Q (n=8,352)	1.12 [1.08, 1.16]	1.09 [1.06, 1.14]	1.02 [0.98, 1.05]
Low SES tract – 1st Q (n=3,574)	1.15 [1.09, 1.20]	1.13 [1.08, 1.19]	1.03 [0.98, 1.08]
Median household income			
High SES tract – 4th Q (n=16,361)	1.00	1.00	1.00
3rd Q (n=7,377)	1.06 [1.03, 1.09]	1.04 [1.01, 1.08]	1.01 [0.98, 1.04]
2nd Q (n=4,331)	1.09 [1.04, 1.13]	1.07 [1.03, 1.11]	1.02 [0.98, 1.05]
Low SES tract – 1st Q (n=3,486)	1.08 [1.02, 1.14]	1.03 [0.98, 1.09]	1.02 [0.97, 1.07]

* CKD identified in EHR was defined as: number of patients who have CKD (eGFR <60ml/min/1.73m^2) documented by ICD9/10 codes in EHR
SES: socioeconomic status, PR: prevalence ratio
Total cohort N= 31,555
Median value of owner-occupied housing units: high SES (4th quartile[Q]):≥$231,300, 3rd Q: $188,100-$231,300, 2nd Q: $165,200-$188,100, low SES(1st Q): <$165,200; %>25 years with a Bachelor's degree or more: high SES (4th quartile[Q]):≥48.1%, 3rd Q: 34.1%-48.1%, 2nd Q: 20.4%-34.1%, low SES(1st Q): <20.4%; Median household income: high SES (4th quartile[Q]):≥$62,343, 3rd Q: $47,379 - $62,343, 2nd Q: $35,935 - $47,379, low SES(1st Q): <$35,935

Model 1: crude
Model 2: age, sex, race, obesity, smoking, insurance status
Model 3: model 2 + history of cardiovascular disease, stroke, cancer, hyperlipidemia, diabetes, hypertension, index eGFR

Table S9. Multilevel regression model for the association of tract level socioeconomic status with angiotensin medication prescription compliance*(***Defining ACEi/ARB prescription compliance based on the ACC/AHA and ADA guidelines)***

	Model 1 (PR, 95% CI)	Model 2 (PR, 95% CI)	Model 3 (PR, 95% CI)
**Median *value of owner-occupied housing units is the measure of SES***			
High SES tract – 4th Q (n=7,804)	1.00	1.00	1.00
3rd Q (n=7,137)	0.99 [0.97, 1.02]	0.99 [0.97, 1.02]	0.99 [0.97, 1.01]
2nd Q (n=2,600)	1.05 [1.01, 1.09]	1.03 [1.00, 1.07]	1.02 [0.98, 1.05]
Low SES tract – 1st Q (n=1,976)	0.99 [0.93, 1.05]	0.98 [0.93, 1.03]	0.96 [0.92, 1.01]
***Percent of residents >25 years with* a Bachelor's degree or more**			
High SES tract (n=5,221) – 4th Q	1.00	1.00	1.00
3rd Q (n=6,561)	1.01 [0.97, 1.05]	1.00 [0.97, 1.04]	0.99 [0.96, 1.03]
2nd Q (n=5,376)	1.04 [1.00, 1.07]	1.03 [0.99, 1.06]	1.01 [0.98, 1.04]
Low SES tract – 1st Q(n=2,359)	1.05 [1.01, 1.09]	1.03 [0.99, 1.06]	1.00 [0.97, 1.04]
Median household income			
High SES tract – 4th Q (n=10,079)	1.00	1.00	1.00
3rd Q (n=4,597)	0.98 [0.95, 1.02]	0.99 [0.94, 1.02]	0.99 [0.96, 1.02]
2nd Q (n=2,728)	0.99 [0.96, 1.03]	1.00 [0.97, 1.03]	0.99 [0.96, 1.03]
Low SES tract – 1st Q (n=2,113)	0.97 [0.94, 1.00]	0.97 [0.94, 1.00]	0.98 [0.95, 1.01]

* Adults (nonpregnant patients) with HTN and CKD (stage 3 or higher, or stage 1 or 2 with UACR >300mg/day) taking angiotensin converting enzyme inhibitor (ACEi) and angiotensin receptor blocker (ARB) or **nonpregnant patients with DM, HTN and albuminuria (UACR >30mg/day)**
Total cohort N= 19,517
SES: socioeconomic status, PR: prevalence ratio of CKD for individual in low SES tract vs. high SES tract
Median value of owner-occupied housing units: high SES (4th quartile[Q]):≥$231,300, 3rd Q: $188,100-$231,300, 2nd Q: $165,200-$188,100, low SES(1st Q): <$165,200; %>25 years with a Bachelor's degree or more: high SES (4th quartile[Q]):≥48.1%, 3rd Q: 34.1%-48.1%, 2nd Q: 20.4%-34.1%, low SES(1st Q): <20.4%; Median household income: high SES (4th quartile[Q]):≥$62,343, 3rd Q: $47,379 - $62,343, 2nd Q: $35,935 - $47,379, low SES(1st Q): <$35,935

Model 1: crude
Model 2: model 1 + age, sex, race, obesity, smoking, insurance status
Model 3: model 2 + history of cardiovascular disease, stroke, cancer, hyperlipidemia, diabetes

Table S10. Multilevel regression model for the association of tract level socioeconomic status with angiotensin medication prescription compliance[1] by sex

	Model 1 (PR, 95% CI)	Model 2 (PR, 95% CI)	Model 3 (PR, 95% CI)
**SEX**			
**Males (n=7,108)**			
**Median *value of owner-occupied housing units is the measure of SES***			
High SES tract – 4th Q (n=3,126)	1.00	1.00	1.00
3rd Q (n=2,492)	0.99 [0.95, 1.04]	0.99 [0.95, 1.03]	0.99 [0.95, 1.03]
2nd Q (n=871)	1.08 [1.03, 1.14]	1.07 [1.03, 1.12]	1.05 [1.01, 1.10]
Low SES tract – 1st Q (n=617)	1.03 [0.94, 1.13]	1.01 [0.92, 1.11]	1.01 [0.92, 1.11]
Percent of residents >25 years with a Bachelor's degree or more			
High SES tract – 4th Q (n=2,105)	1.00	1.00	1.00
3rd Q (n=2,369)	1.02 [0.97, 1.07]	1.01 [0.96, 1.07]	1.01 [0.96, 1.06]
2nd Q (n=1,853)	1.03 [0.98, 1.09]	1.02 [0.97, 1.07]	1.01 [0.96, 1.06]
Low SES tract – 1st Q (n=780)	1.08 [1.02, 1.14]	1.05 [0.99, 1.11]	1.04 [0.98, 1.09]
**Females (n=9,668)**			
**Median *value of owner-occupied housing units is the measure of SES***			
High SES tract – 4TH Q (n=3,699)	1.00	1.00	1.00
3rd Q (n=3,652)	0.99 [0.96, 1.03]	0.99 [0.95, 1.02]	0.99 [0.94, 1.03]
2nd Q (n=1,325)	1.01 [0.89, 1.02]	0.99 [0.94, 1.04]	0.98 [0.94, 1.03]
Low SES tract – 1st Q (n=990)	0.95 [0.89, 1.02]	0.94 [0.88, 1.02]	0.93 [0.87, 0.99]
***Percent of >25 years with* a Bachelor's degree or more**			
High SES tract – 4th Q (n=2,495)	1.00	1.00	1.00
3rd Q (n=3,299)	0.99 [0.95, 1.04]	0.98 [0.94, 1.03]	0.98 [0.94, 1.02]
2nd Q (n=2,707)	1.03 [0.98, 1.07]	1.03 [0.99, 1.07]	1.01 [0.97, 1.06]
Low SES tract – 1st Q (n=1,165)	1.02 [0.97, 1.07]	0.99 [0.95, 1.05]	0.98 [0.93, 1.03]

1-Adults (nonpregnant patients) with hypertension and chronic kidney disease (stage 3 or higher, or stage 1 or 2 with UACR >300mg/day) should be taking ACEi/ARB. ACEI/ARB prescription compliance: yes if recommended ACEI/ARB prescribed;
PR: prevalence ratio; Median value of owner-occupied housing units: high SES (4th quartile[Q]):≥$231,300, 3rd Q: $188,100-$231,300, 2nd Q: $165,200-$188,100, low SES(1st Q): <$165,200; %>25 years with a Bachelor's degree or more: high SES (4th quartile[Q]):≥48.1%, 3rd Q: 34.1%-48.1%, 2nd Q: 20.4%-34.1%, low SES(1st Q): <20.4

Model 1: crude; **Model 2:** age, race, obesity, smoking, insurance status; **Model 3:** model 2 + history of cardiovascular disease, stroke, cancer, hyperlipidemia, diabetes/ **We removed variables we stratified by from models**

Table S11. Multilevel regression model for the association of tract level socioeconomic status with angiotensin medication prescription compliance by race

	Model 1 (PR, 95% CI)	Model 2 (PR, 95% CI)	Model 3 (PR, 95% CI)
**RACE**			
***Percent of >25 years with* a Bachelor's degree or more**			
**Black (n=885)**			
High SES tract – 4th Q (n=153)	1.00	1.00	1.00
3rd Q (n=247)	1.14 [0.99, 1.31]	1.15 [1.00, 1.33]	1.14 [1.00, 1.31]
2nd Q (n=276)	1.05 [0.91, 1.21]	1.06 [0.91, 1.24]	1.05 [0.91, 1.22]
Low SES tract – 1st Q (n=209)	0.98 [0.82, 1.15]	0.99 [0.84, 1.18]	0.97 [0.82, 1.14]
**Non-Black (n=15,891)**			
Percent of >25 years with a Bachelor's degree or more			
High SES tract – 4TH Q (n=4,447)	1.00	1.00	1.00
3rd Q (n=5,421)	0.99 [0.96, 1.04]	0.99 [0.95, 1.03]	0.98 [0.95, 1.02]
2nd Q (n=4,284)	1.03 [0.99, 1.07]	1.02 [0.99, 1.06]	1.01 [0.98, 1.05]
Low SES tract – 1st Q (n=1,736)	1.05 [1.00, 1.10]	1.03 [0.99, 1.08]	1.02 [0.97, 1.06]

1-Adults (nonpregnant patients) with hypertension and chronic kidney disease (stage 3 or higher, or stage 1 or 2 with UACR >300mg/day) should be taking ACEi/ARB. ACEI/ARB prescription compliance: yes if recommended ACEI/ARB prescribed;
PR: prevalence ratio; Median value of owner-occupied housing units: high SES (4th quartile[Q]):≥$231,300, 3rd Q: $188,100-$231,300, 2nd Q: $165,200-$188,100, low SES(1st Q): <$165,200; %>25 years with a Bachelor's degree or more: high SES (4th quartile[Q]):≥48.1%, 3rd Q: 34.1%-48.1%, 2nd Q: 20.4%-34.1%, low SES(1st Q): <20.4

Model 1: crude; **Model 2:** age, race, obesity, smoking, insurance status; **Model 3:** model 2 + history of cardiovascular disease, stroke, cancer, hyperlipidemia, diabetes/ **We removed variables we stratified by from models**

Table S12. Multilevel regression model for the association of tract level socioeconomic status with CKD identified in the EHR[1] for the identified effect modifiers

	Model 1 (PR, 95% CI)	Model 2 (PR, 95% CI)	Model 3 (PR, 95% CI)
**Hypertension**			
**HAVE HYPERTENSION (n= 19,627)**			
**Median *value of owner-occupied housing units is the measure of SES***			
High SES tract – 4th Q (n=7,938)	1.00	1.00	1.00
3rd Q (n= 7,203)	1.05 [1.02, 1.08]	1.05 [1.02, 1.07]	1.02 [0.97, 1.05]
2nd Q (n=2,557)	1.08 [1.03, 1.13]	1.06 [1.02, 1.11]	1.02 [0.98, 1.06]
Low SES tract – 1st Q (n=1,929)	1.08 [1.04, 1.13]	1.05 [1.01, 1.09]	1.00 [0.97, 1.03]
Percent of >25 years with a Bachelor's degree or more			
High SES tract – 4th Q (n=5,335)	1.00	1.00	1.00
3rd Q (n=6,658)	1.04 [1.01, 1.07]	1.04 [1.00, 1.12]	1.01 [0.98, 1.06]
2nd Q (n=5,371)	1.05 [1.02, 1.08]	1.04 [1.01, 1.08]	1.00 [0.97, 1.03]
Low SES tract – 1st Q (n=2,263)	1.08 [1.04, 1.14]	1.07 [1.02, 1.12]	1.02 [0.98, 1.06]
**HAVE NO HYPERTENSION (n=5,470)**			
**Median *value of owner-occupied housing units is the measure of SES***			
High SES tract – 4th Q (n=2,673)	1.00	1.00	1.00
3rd Q (n=1,737)	1.19 [1.06, 1.33]	1.19 [1.06, 1.34]	1.11 [0.99, 1.25]
2nd Q (n=607)	1.31 [1.12, 1.54]	1.31 [1.11, 1.54]	1.14 [0.98, 1.32]
Low SES tract – 1st Q (n=453)	1.30 [1.08, 1.56]	1.34 [1.11, 1.62]	1.22 [1.01, 1.46]
***Percent of >25 years with* a Bachelor's degree or more**			
High SES tract – 4th Q (n=1,838)	1.00	1.00	1.00
3rd Q (n=1,792)	1.11 [0.98, 1.26]	1.14 [0.99, 1.30]	1.07 [0.95, 1.22]
2nd Q (n=1,302)	1.28 [1.11, 1.46]	1.27 [1.10, 1.46]	1.15 [1.00, 1.31]
Low SES tract – 1st Q (n=538)	1.28 [1.06, 1.53]	1.34 [1.10, 1.63]	1.17 [0.96, 1.42]

1-CKD identified in EHR was defined as: number of patients who have CKD (eGFR <60ml/min/1.73m^2) documented by ICD9/10 codes in EHR / number of patients with CKD x 100%

SES: socioeconomic status, PR: prevalence ratio

Median value of owner-occupied housing units: high SES (4th quartile[Q]):≥$231,300, 3rd Q: $188,100-$231,300, 2nd Q: $165,200-$188,100, low SES(1st Q): <$165,200; %>25 years with a Bachelor's degree or more: high SES (4th quartile[Q]):≥48.1%, 3rd Q: 34.1%-48.1%, 2nd Q: 20.4%-34.1%, low SES(1st Q): <20.4%; Median household income: high SES (4th quartile[Q]):≥$62,343, 3rd Q: $47,379 - $62,343, 2nd Q: $35,935 - $47,379, low SES(1st Q): <$35,935

Model 1: crude
Model 2: age, sex, race, obesity, smoking, insurance status
Model 3: model 2 + history of cardiovascular disease, stroke, cancer, hyperlipidemia, diabetes, hypertension, index eGFR

- We removed variables we stratified by from models

Table S12. (continued)

	Model 1 (PR, 95% CI)	Model 2 (PR, 95% CI)	Model 3 (PR, 95% CI)
**Diabetes**			
**HAVE DIABETES (n=7,763)**			
**Median *value of owner-occupied housing units is the measure of SES***			
High SES tract – 4th Q (n=2,842)	1.00	1.00	1.00
3rd Q (n=2,895)	1.03 [0.99, 1.06]	1.02 [0.99, 1.05]	1.01 [0.98, 1.04]
2nd Q (n=1,121)	1.06 [1.02, 1.11]	1.05 [1.00, 1.09]	1.03 [0.99, 1.07]
Low SES tract – 1st Q (n=905)	1.00 [0.95, 1.06]	0.98 [0.93, 1.04]	0.98 [0.93, 1.04]
Median household income			
High SES tract – 4th Q (n=3,859)	1.00	1.00	1.00
3rd Q (n=1,868)	1.01 [0.98, 1.05]	1.01 [0.97, 1.05]	0.99 [0.96, 1.03]
2nd Q (n=1,159)	1.01 [0.97, 1.05]	1.00 [0.96, 1.04]	0.99 [0.95, 1.03]
Low SES tract – 1st Q (n=877)	1.01 [0.96, 1.06]	0.97 [0.93, 1.02]	0.97 [0.93, 1.02]
**HAVE NO DIABETES (n=17,334)**			
**Median *value of owner-occupied housing units is the measure of SES***			
High SES tract – 4th Q (n=7,769)	1.00	1.00	1.00
3rd Q (n=6,045)	1.11 [1.07, 1.16]	1.09 [1.05, 1.13]	1.04 [1.00, 1.08]
2nd Q (n=2,043)	1.12 [1.04, 1.21]	1.09 [1.02, 1.17]	1.04 [0.98, 1.10]
Low SES tract – 1st Q (n=1,477)	1.16 [1.09, 1.24]	1.13 [1.07, 1.19]	1.03 [0.98, 1.09]
Median household income			
High SES tract – 4th Q (n=9,245)	1.00	1.00	1.00
3rd Q (n=3,989)	1.09 [1.04, 1.14]	1.05 [1.01, 1.09]	1.04 [0.98, 1.10]
2nd Q (n=2,232)	1.12 [1.07, 1.19]	1.08 [1.03, 1.13]	1.03 [0.98, 1.08]
Low SES tract – 1st Q (n=1,868)	1.09 [1.02, 1.17]	1.06 [0.99, 1.13]	1.04 [0.98, 1.06]

Table S12. (continued)

	Model 1 (PR, 95% CI)	Model 2 (PR, 95% CI)	Model 3 (PR, 95% CI)
**Race**			
**Blacks (n=1,130)**			
**Median *value of owner-occupied housing units is the measure of SES***			
High SES tract – 4th Q (n=245)	1.00	1.00	1.00
3rd Q (n=383)	1.06 [0.98, 1.14]	1.07 [0.99, 1.15]	1.01 [0.94, 1.07]
2nd Q (n=210)	1.01 [0.92, 1.11]	1.00 [0.90, 1.12]	0.96 [0.88, 1.06]
Low SES tract – 1st Q (n=292)	0.97 [0.89, 1.06]	0.97 [0.89, 1.06]	0.94 [0.87, 1.02]
**Non-Blacks (n=23,967)**			
**Median *value of owner-occupied housing units is the measure of SES***			
High SES tract – 4th Q (n=10,366)	1.00	1.00	1.00
3rd Q (n=8,557)	1.10 [1.06, 1.14]	1.07 [1.04, 1.10]	1.03 [1.00, 1.05]
2nd Q (n=2,054)	1.13 [1.07, 1.20]	1.09 [1.04, 1.14]	1.04 [0.99, 1.09]
Low SES tract – 1st Q (n=2,083)	1.12 [1.06, 1.19]	1.09 [1.04, 1.14]	1.03 [1.00, 1.08]

Table S13. Description of cohort excluded from analyses

	**Cohort of excluded adults (1 outpatient clinic visit from 7/1/2017 to 12/31/2018 & no inpatient/outpatient creatinine & have address available)**<br>N= 104,860
**Individual level characteristics**	
Age, mean (SD)	40.5 ± 17.0
Male, n(%)	44,447 (43%)
Black	8,442 (8%)
Ever Smoker, n(%)	25173 (24%)
**Vitals**	
Systolic BP, mmHg	121.4 ± 14.0
Diastolic BP, mmHg	75.4 ± 9.8
**Medical History**	
Hypertension, n(%)	2793 (3%)
Diabetes, n(%)	1663 (2%)
Obese (BMI≥ 30 kg/m^2)[1], n(%)	20306 (27%)
Cardiovascular disease, n(%)	557 (0.5%)
Stroke, n(%)	211 (0.2%)
Hyperlipidemia, n(%)	3081 (3%)
Cancer, n(%)	1291 (1%)
**Median value of owner occupied housing units**	
Q1: < $165,200	10848 (10%)
Q2: $165,200 - $188,100	13999 (13%)
Q3: $188,100 - $231,300	31724 (30%)
Q4: ≥ $231,300	48261 (46%)
**% of Residents > 25 years with complete college education**	
Q1: < 20.4%	12109 (12%)
Q2: 20.4% - 34.1%	25480 (24%)
Q3: 34.1% - 48.1%	29915 (29%)
Q4: ≥ 48.1%	37337 (36%)
**Median household income**	
Q1: <$35,935	15829 (15%)
Q2: $35,935 - $47,379	20948 (20%)
Q3: $47,379 - $62,343	27410 (26%)
Q4: ≥ $62,343	40673 (39%)

Cardiovascular disease includes congestive heart failure, acute myocardial infarction, ischemic heart disease, and peripheral vascular disease

1- Body Mass Index (BMI) was missing in 29% of the excluded cohort

Table S14. Comparison of Fairview patients to the 7-county Minneapolis/St Paul metropolitan area

	Fairview population (included in our analysis) to assess ACEI/ARB prescription compliance n=16,776	Fairview population (included in our analysis) to assess UACR measurement performance and CKD identification n=25,095	Census data (7 county metro area)
Median Age[1]	55	56	36*
% Black	5%	5%	8%
% Male	42%	41%	49%
%Medicaid[2]	7%	7%	7%
Number of census tracts	666	666	704
Population by County, n(%)			
Anoka	2,677 (16%)	3,773 (15%)	331,649 (12%)
Carver	180 (1%)	275 (1%)	91,355 (3%)
Dakota	3,795 (22%)	5,807 (23%)	399,443 (14%)
Hennepin	7,120 (42%)	10,506 (42%)	1,158,039 (40%)
Ramsey	1,742 (10%)	2,718 (11%)	510,885 (18%)
Scott	699 (4%)	1,101 (4%)	130,689 (5%)
Washington	563 (3%)	917 (4%)	238,721 (8%)
Total population	*16,776*	*25,097*	*2,860,781*

[1]Median age in Fairview population before excluding patients <18 years
[2]Medicaid in Fairview population is calculated for those ≥ 18 years with Medicaid coverage
Medicaid coverage (Medicaid or other means-tested public coverage) for census data include individuals ≥18 years with coverage through Medicaid, Medical Assistance or any kind of government assistance plan for those with low incomes or a disability from 2012 American Community Survey: 5-Year Data [2008-2012].
*Census data is not restricted to adults >18 years. Median age here is of all the population.

ACEi/ARB: angiotensin converting enzyme inhibitor/angiotensin receptor blocker
UACR: urine albumin to creatinine ratio
CKD: chronic kidney disease
